# Research hotspots and trends on spinal cord stimulation for pain treatment: a two-decade bibliometric analysis

**DOI:** 10.3389/fnins.2023.1158712

**Published:** 2023-05-25

**Authors:** Sheng Yang, Sen Zhong, Yunshan Fan, Yanjie Zhu, Ningze Xu, Yue Liao, Guoxin Fan, Xiang Liao, Shisheng He

**Affiliations:** ^1^Department of Orthopedic, Spinal Pain Research Institute, Shanghai Tenth People’s Hospital, Tongji University School of Medicine, Shanghai, China; ^2^Shanghai Tongji Hospital, Tongji University School of Medicine, Shanghai, China; ^3^Shanghai Tenth People’s Hospital, Tongji University School of Medicine, Shanghai, China; ^4^Department of Pain Medicine, Huazhong University of Science and Technology Union Shenzhen Hospital, Shenzhen, China; ^5^Guangdong Key Laboratory for Biomedical Measurements and Ultrasound Imaging, National-Regional Key Technology Engineering Laboratory for Medical Ultrasound, School of Biomedical Engineering, Shenzhen University Medical school, Shenzhen, China; ^6^Department of Spine Surgery, Third Affiliated Hospital, Sun Yat-sen University, Guangzhou, China

**Keywords:** spinal cord stimulation, pain treatment, bibliometric, research trends, CiteSpace, VOSviewer, bibliometrix

## Abstract

**Background:**

Chronic pain poses a significant social burden. Spinal cord stimulation (SCS) is considered to be the most promising treatment for refractory pain. The aim of this study was to summarize the current research hotspots on SCS for pain treatment during the past two decades and to predict the future research trends by bibliometric analysis.

**Methods:**

The literature over the last two decades (2002–2022) which was related to SCS in pain treatment was obtained from the Web of Science Core Collection. Bibliometric analyses were conducted based on the following aspects: (1) Annual publication and citation trends; (2) Annual publication changes of different publication types; (3) Publications and citations/co-citations of different country/institution/journal/author; (4) Citations/co-citation and citation burst analysis of different literature; and (5) Co-occurrence, cluster, thematic map, trend topics, and citation burst analysis of different keywords. (6) Comparison between the United States and Europe. All analyses were performed on CiteSpace, VOSviewer, and R bibliometrix package.

**Results:**

A total of 1,392 articles were included in this study, with an increasing number of publications and citations year by year. The most highly published type of literature was clinical trial. United States was the country with the most publications and citations; Johns Hopkins University was the institution with the most publications; NEUROMODULATION published the most papers; the most published author was Linderoth B; and the most cited paper was published in the PAIN by Kumar K in 2007. The most frequently occurring keywords were “spinal cord stimulation,” “neuropathic pain,” and “chronic pain,” etc.

**Conclusion:**

The positive effect of SCS on pain treatment has continued to arouse the enthusiasm of researchers in this field. Future research should focus on the development of new technologies, innovative applications, and clinical trials for SCS. This study might facilitate researchers to comprehensively understand the overall perspective, research hotspots, and future development trends in this field, as well as seek collaboration with other researchers.

## Introduction

Pain is an unpleasant sensory and emotional experience (Raja et al., 2020). Acute pain has a physiological significance, whereas chronic pain primarily causes human suffering (Treede et al., 2019; Cohen et al., 2021). It has been reported that 20.5% people from the United States are suffering from chronic pain (Yong et al., 2022), whereas estimates in the United Kingdom range from 13 to 50%, which prevents them from working (Mills et al., 2019), affects them mentally, causes numerous psychological problems, etc., and imposes a huge economic burden on individuals and society (Breivik et al., 2013; Cohen et al., 2021). According to one study, patients with pain pay an additional $261 per year in medical costs, which causes a socio-economic toll of over $29.9 billion (Gaskin and Richard, 2012).

Pain can be treated in several ways, including pharmacological, interventional, surgical, exercise, and psychological treatments (Cohen et al., 2021). In recent years, various physiological treatments, such as massage, reiki, mindfulness meditation, complementary and alternative therapies, and music therapy, have also shown to be effective (Furlan et al., 2015; Garza-Villarreal et al., 2017; Hilton et al., 2017; Billot et al., 2019; Paley and Johnson, 2020). However, for refractory chronic pain, these treatments have limited effects, and the most promising treatment is currently neuromodulation, such as spinal cord stimulation (SCS)(Pirvulescu et al., 2022). After Melzack, R. proposed the gate-control theory of pain in 1965, SCS was then first applied to the clinical treatment of chronic pain in 1967, achieving satisfactory pain relief (Melzack and Wall, 1965; Shealy et al., 1967). The use of SCS therapy has grown rapidly over the past two decades, and the market for SCS instruments is expected to reach $2.8 billion by 2025 (Knotkova et al., 2021).

Bibliometric analysis is a scientific method for assessing the impact and development of a certain research field and is often used to have a deep understanding of the research field (Keathley-Herring et al., 2016). The literature on SCS for pain treatment has been increasing over the past two decades. However, there are no relevant studies that provide a systematic summary of the developments in this field. The aim of this study is to generalize the development of this field over the past two decades through bibliometric analysis and to predict future research hotspots and trends.

## Methods

### Data collection and retrieval strategy

**Figure 1:**
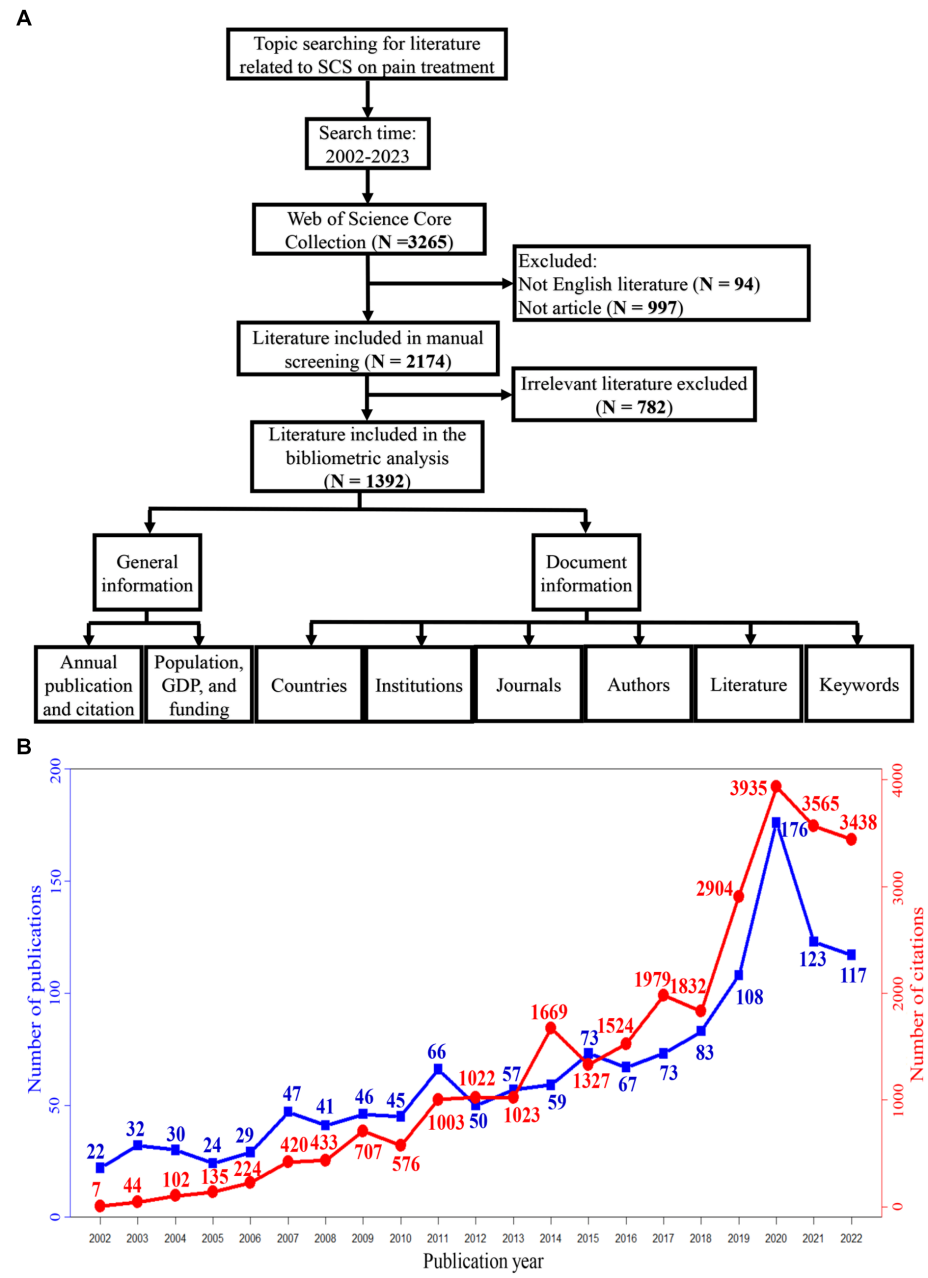
**(A)** The screening process of selected literature in this study. **(B)** Annual trends of publications and citations.

We searched the literature from the Web of Science Core Collection (WOSCC) with the strategy formula (TS = ((“spinal stimulation” OR “spinal cord stimulation” OR “epidural spinal stimulation” OR “epidural electrical stimulation” OR “transcutaneous spinal stimulation” OR “intraspinal stimulation” OR “intra-spinal stimulation” OR “intraspinal electrical stimulation” OR “intra-spinal electrical stimulation” OR “intraspinal microstimulation” OR “intraspinal micro-stimulation” OR “intra-spinal microstimulation” OR “intra-spinal micro-stimulation” OR “spinal cord electrical stimulation”))) AND ((TS = (pain*))) for the period from January 1, 2002, to March 28, 2023. A total of 3,265 papers were obtained. After removing 94 papers that were not published in English and 997 papers that were not published as article, 2,174 papers were included. Then two people reviewed the literature separately to decide whether the literature belonged to research of SCS on pain, when there was disagreement, a third person reviewed it again and made the final decision. Finally, 1,392 papers were identified and included in the analysis. Figure 1A showed the flow chart of this study.

### Analysis method

The bibliometric analyses was performed using CiteSpace (version 6.1.R2), VOSviewer (version 1.6.11.0), and bibliometrix R package (version 3.0.4). Our analyses included the following aspects.

1. Annual trends of publication/citation. Using the R ggplot2 package, we overviewed the development of the field.
2. Analysis of publication types. We classified the literature included into four research types refer to PubMed’s classification, including basic research, case report, clinical trial, and randomized controlled trial (RCT). Papers not recorded in PubMed were manually reviewed and classified. Finally, we analyzed the annual changes of different publication types using the R ggplot2 package.
3. Analysis of countries/regions and institutions. The publications of different countries/regions and institutions were analyzed using CiteSpace, while the citation analyses were performed on VOSviewer, which provided the insight into the research status of different countries/regions and institutions. In the analysis data, if A country published a paper, which cited the paper published by B country, representing the country A and B would form a citation relationship, and the A node was connected to the B node in the figure. The more citations a country/institution has, the more likely it has published some significant findings.
4. Analysis of journals and authors. The publications of different journals and authors were summarized, while their citation and co-citation networks were created using VOSviewer. The citation networks were formed similarly to the countries’. In the analysis data, if a paper published on journal A, which cited the paper published on journal B and the paper published on journal C at the same time, there would be a co-citation relationship between journal B and C, and the co-citation counts for B and C were both increased by one. The journal/author acquired more attention when it had more co-citation counts.
5. Analysis of literature. The co-citation network and citation burst analysis of different literature were conducted by VOSviewer and CiteSpace respectively, to access the significant achievements and research hotspots in the field.
6. Analysis of keywords. The keyword co-occurrence and cluster analysis were performed at 5-year intervals using CiteSpace. Besides, the factorial analysis, trend topics, and theme map analysis were conducted by the R bibliometrix package. Citation burst analysis was conducted using CiteSpace. All of these aimed to find out the research hotspots and future trends in the field.
7. Comparisons between the United States and the Europe. We analyzed the representative authors and institutions as well as their research themes of the USA and the Europe using bibliometrix R package. In addition, the evolution of their research themes were also analyzed.

## Results

### Publication and citation trends

As shown in Figure 1B, the number of annual publications in this field increased from 22 in 2002 to 176 in 2020, with an overall increasing trend (blue curve). The citation trend (red curve) showed an increasing number of annual citations, from 7 in 2002 to 3,935 in 2020, while the latter 2 years began to decrease.

### Publication type of literature

**Figure 2:**
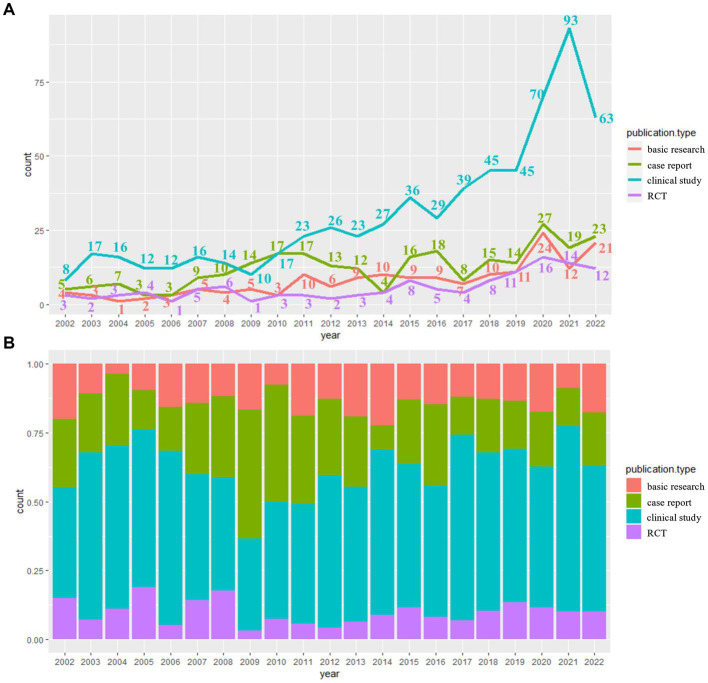
Annual changes in different publication types. **(A)** The annual change of different publication types presented by line chart. **(B)** The annual change of different publication types presented by histogram.

As shown in Figure 2, the most published type was clinical trial, with a total of 641 papers, followed by the case report of 260 papers, with an upward trend in recent years, and the remaining types had a relatively slow growth.

### Country/region and institution analysis

**Figure 3:**
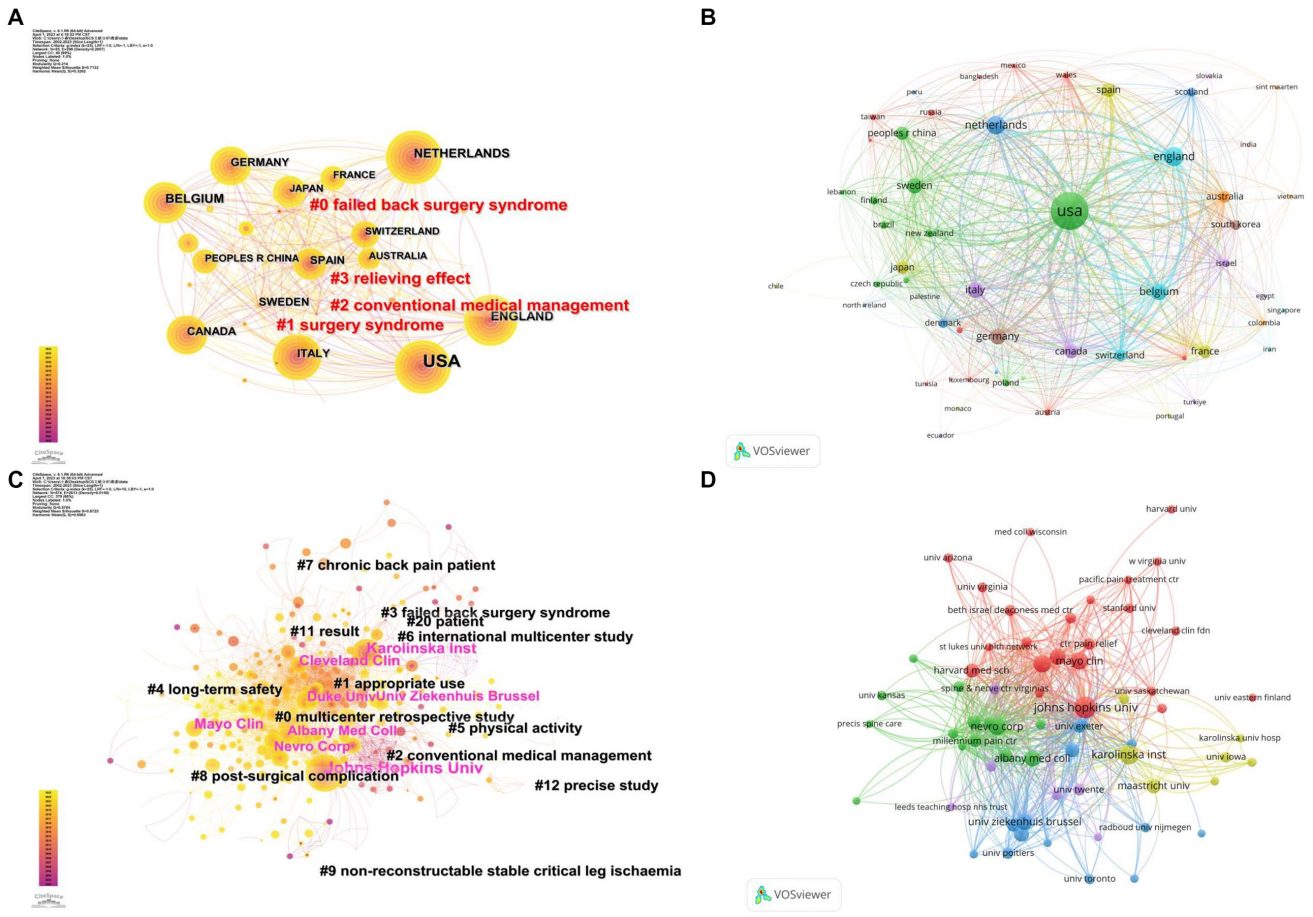
The analysis of countries/regions and institutions. **(A)** Network map of collaborations among different countries/regions and cluster analysis by CiteSpace. **(B)** Network map of citations among different countries/regions by VOSviewer. **(C)** Network map of collaborations among different institutions and cluster analysis by CiteSpace. **(D)** Network map of citations among different institutions by VOSviewer.

The publication analysis of different countries/regions was presented in Figure 3A, where the nodes represented different countries/regions, the node size represented the publication volume, and the connecting lines represented collaborations among countries. Based on the publication volume, the top 10 countries with the most publications were summarized in Table 1: United States (749), England (138), Netherlands (120), Belgium (92), Germany (81). The publication volumes of different countries were demonstrated on a world map in Supplementary Figure S1.

**Table 1:** The top 10 countries/regions and institutions with the most publications and their citations.

Ranking	Country/Region	Papers	Citations	Citations per paper	Institution	Papers	Citations	Citations per paper
1	United States	749	15,744	21.02	Johns Hopkins University	60	3,300	55.00
2	England	138	5,552	40.23	Albany Medical Center	59	612	10.37
3	Netherlands	120	3,763	31.36	Karolinska Institute	58	2,825	48.71
4	Belgium	92	3,800	41.30	Cleveland Clinic	47	1,506	32.04
5	Germany	81	1724	21.28	Poitiers University Hospital	42	326	7.76
6	Italy	79	2,778	35.16	Mayo clinic	38	385	10.13
7	Sweden	75	2,874	38.32	Harvard University	35	456	13.03
8	Spain	58	2,301	39.67	Duke University	33	746	22.61
9	Canada	56	3,260	58.21	Nevro Corporation	32	1,239	38.72
10	Peoples R China	53	417	7.87	Universitair Ziekenhuis Brussel	32	235	7.34

The publication analysis of different institutions was shown in Figure 3C. A total of 1,547 institutions were involved in this study, and the top 10 most published institutions were shown in Table 1: Johns Hopkins University (Hasoon et al., 2023), followed by Albany Medical Center (Kapural and Calodney, 2022), Karolinska Institute (Grider et al., 2016), Cleveland Clinic (Kumar et al., 2002), and Poitiers University Hospital (Paul et al., 2017).

The citation networks of different countries and institutions were shown in Figures 3B,D. The node size represented the citation counts, and the connecting lines represented the citation relationships between nodes. The top 10 countries and institutions were summarized in Supplementary Table S1, where the top five countries were USA (15744), England (5552), Belgium (3800), Netherlands (3763), and Canada (3260), and the top five institutions were Johns Hopkins University (3300), Karolinska Institute (2825), University of Exeter (2318), Regina general hospital (1710), and Cleveland Clinic (1506).

The population and GDP of the top 10 countries with the most publications were summarized in Supplementary Table S2 based on World Bank data 2021. The top 10 funding agencies for the output of research in this field were summarized in Supplementary Table S3. In addition, cluster analyses were conducted for countries and institutions, while the top five countries and institutions in each cluster were listed in Supplementary Tables S4, S5.

### Journal analysis

**Figure 4:**
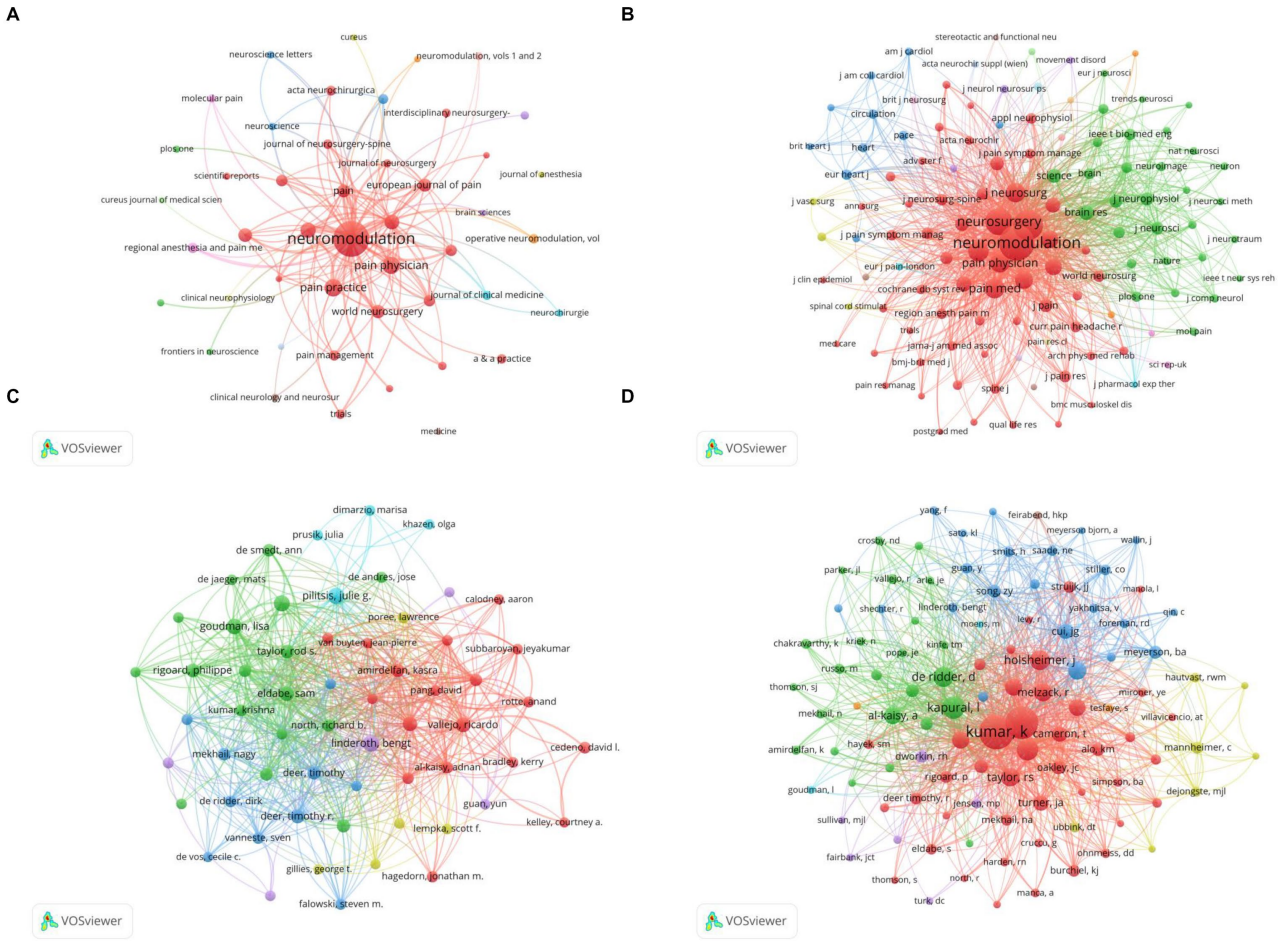
The analysis of journals and authors by VOSviewer. **(A)** Network map of publications for different journals. **(B)** Network map of co-citations among different journals. **(C)** Network map of publications for different authors. **(D)** Network map of co-citations among different authors.

All the papers included in the analysis were published in 287 journals. To highlight the important journals, we selected journals with more than 5 publications for the analysis. The citation network was shown in Figure 4A while the co-citation network was in Figure 4B. Node size represented the number of publications and co-citations respectively, and the connecting lines represented the citation and co-citation relationships between nodes. The top 10 journals with the most publications or co-citations were listed in Table 2. The journal with the highest number of papers was NEUROMODULATION (394, IF3.025, Q3), followed by PAIN PRACTICE (61, IF3.079, Q3), and PAIN PHYSICIAN (58, IF4.396, Q2). The journal with the highest number of co-citations was NEUROMODULATION (5,622, IF3.025, Q3), PAIN (3,523, IF7.926, Q1), followed by and NEUROSURGERY (2,625, IF5.315, Q1).

**Table 2:** The top 10 journals with the most publications and co-citations.

Ranking	Journal	Frequency	JCR category	Category rank 2021	Category quartile 2021	IF 2021
1	Neuromodulation	394	Clinical neurology; medicine research and experimental	131/212;96/139	Q3;Q3	3.025
2	Pain practice	61	Anesthesiology; clinical neurology	20/34;128/212	Q3;Q3	3.079
3	Pain physician	58	Anesthesiology; clinical neurology	11/34;77/212	Q2;Q2	4.396
4	Pain medicine	42	Anesthesiology; medicine, general and internal	16/34;65/172	Q2;Q2	3.637
5	Neurosurgery	40	Clinical neurology; surgery	52/212;26/213	Q1;Q1	5.315
6	Journal of pain research	32	Clinical neurology	139/212	Q3	2.832
7	World neurosurgery	30	Clinical neurology; surgery	170/212;124/213	Q4;Q3	2.21
8	Pain	29	Anesthesiology; clinical neurology; neurosciences	5/34;21/212;33/275	Q1;Q1;Q1	7.926
9	European journal of pain	28	Anesthesiology; clinical neurology; neurosciences	15/34; 103/212; 153/275	Q2;Q2;Q3	3.651
10	Stereotactic and functional neurosurgery	21	Neuroimaging; neurosciences; surgery	13/14;253/275;160/213	Q4;Q4;Q4	1.643
Ranking	Co-cited journals	Frequency	JCR category	Category Rank 2021	Category Quartile 2021	IF 2021
1	Neuromodulation	5,622	Clinical neurology; medicine, research and experimental	131/212;96/139	Q3;Q3	3.025
2	Pain	3,523	Anesthesiology; clinical neurology; neurosciences	5/34;21/212;33/275	Q1;Q1;Q1	7.926
3	Neurosurgery	2,625	Clinical neurology; surgery	52/212;26/213	Q1;Q1	5.315
4	Spine	1,254	Clinical neurology; orthopedics	117/212;29/86	Q3;Q2	3.269
5	Pain medicine	1,181	Anesthesiology; medicine, general and internal	16/34;65/172	Q2;Q2	3.637
6	Journal of neurosurgery	1,045	Clinical neurology; surgery	45/212;23/213	Q1;Q1	5.526
7	Pain physician	1,044	Anesthesiology; clinical neurology	11/34;77/212	Q2;Q2	4.396
8	Pain practice	869	Anesthesiology; clinical neurology	20/34;128/212	Q3;Q3	3.079
9	European journal of pain	801	Anesthesiology; clinical neurology; neurosciences	15/34; 103/212; 153/275	Q2;Q2;Q3	3.651
10	Anesthesiology	730	Anesthesiology	4/34	Q1	9.198

### Author analysis

To better show the citation and co-citation relationships among authors, we only selected authors with more than 10 publications for analysis, and the citation relationships were shown in Figure 4C. The co-citation network of authors with more than 200 co-citations was shown in Figure 4D. The node sizes in Figures 4C,D represented the number of publications and co-citations, respectively. The top 10 authors with the highest number of publications or co-citations were summarized in Table 3. The author with the highest number of publications was Linderoth B, with 53 publications, and Kumar K was the author with the highest number of co-citations (1395).

**Table 3:** The top 10 authors with the most publications and co-citations.

Ranking	Author	Frequency	Country/Region	Co-cited author	Frequency	Country/Region
1	Linderoth, Bengt	53	United States	Kumar, Krishna	1,395	Canada
2	Deer, Timothy R.	45	England	Deer, Timothy R.	606	United States
3	Pilitsis, Julie G.	43	Belgium	Kapural, Leonardo	554	United States
4	Moens, Maartens	36	Netherlands	Kemler, Marius A.	492	Netherlands
6	Goudman, Lisa	32	United States	Woolf, Clifford J.	453	United States
5	Kapural, Leonardo	29	Sweden	De Ridder, Dirk	433	Belgium
7	Joosten, Elbert A. J.	29	Belgium	Linderoth, Bengt	415	Sweden
9	Vallejo, Ricardo	28	United States	Melzack, Ronald	342	Canada
8	Eldabe, Sam	27	United States	Al-kaisy, Adnan	323	England
10	North, Richard B.	26	United States	Barolat, Giancarlo	313	United States

### Literature analysis

**Figure 5:**
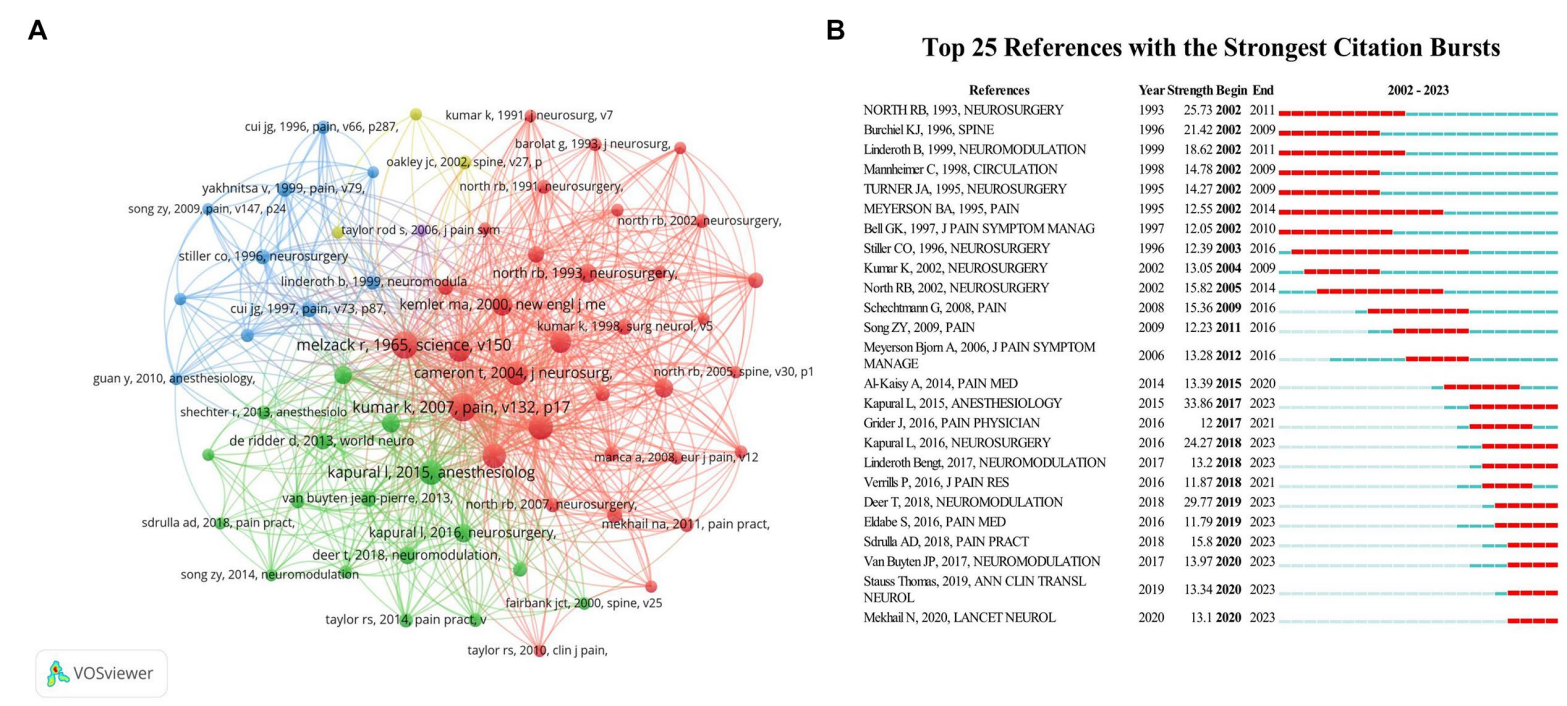
The analysis of literature. **(A)** Network map of co-citations among different literature by VOSviewer. **(B)** The top 25 articles with the strongest citation bursts by CiteSpace.

The co-citation network of literature was demonstrated in Figure 5A. The node size represented the number of co-citations, and the line between nodes represented the co-citation relationship between nodes. The top 10 cited and co-cited papers were summarized in Table 4. Interestingly, the most cited and co-cited paper were the same one “Spinal Cord Stimulation Versus Conventional Medical Management For Neuropathic Pain: A Multicentre Randomised Controlled Trial In Patients With Failed Back Surgery Syndrome,” which was published on PAIN by Kumar K in 2007, with a total of 671 citations and 311 co-citations.

**Table 4:** The top 10 articles with the most citations and co-citations.

Ranking	Literature	Cited counts	Journal	First author (year)	Country/Region
1	Spinal Cord Stimulation Versus Conventional Medical Management For Neuropathic Pain: A Multicentre Randomised Controlled Trial In Patients With Failed Back Surgery Syndrome	671	Pain	Kumar, Krishna (2007)	Canada
2	Spinal Cord Stimulation Versus Repeated Lumbosacral Spine Surgery For Chronic Pain: A Randomized, Controlled Trial	531	Neurosurgery	North, RB (2005)	United States
3	Novel 10-Khz High-Frequency Therapy (HF10 Therapy) Is Superior To Traditional Low-Frequency Spinal Cord Stimulation For The Treatment Of Chronic Back And Leg Pain	444	Anesthesiology	Kapural, Leonardo (2015)	United States
4	The Effects Of Spinal Cord Stimulation In Neuropathic Pain Are Sustained: A 24-Month Follow-Up Of The Prospective Randomized Controlled Multicenter Trial Of The Effectiveness Of Spinal Cord Stimulation	423	Neurosurgery	Kumar, Krishna (2008)	Canada
5	Spinal Cord Stimulation In Treatment Of Chronic Benign Pain: Challenges In Treatment Planning And Present Status, A 22-Year Experience	285	Neurosurgery	Kumar, Krishna (2006)	Canada
6	Comparison Of 10-Khz High-Frequency And Traditional Low-Frequency Spinal Cord Stimulation For The Treatment Of Chronic Back And Leg Pain: 24-Month Results From A Multicenter, Randomized, Controlled Pivotal Trial	259	Neurosurgery	Kapural, Leonardo (2016)	United States
7	Effect Of Spinal Cord Stimulation For Chronic Complex Regional Pain Syndrome Type I: Five-Year Final Follow-Up Of Patients In A Randomized Controlled Trial	238	Journal of neurosurgery	Kemler, Marius A. (2008)	Netherlands
8	Success Using Neuromodulation With BURST (SUNBURST) Study: Results From a Prospective, Randomized Controlled Trial Using a Novel Burst Waveform	226	Neuromodulation	Deer, Timothy (2018)	United States
9	Burst Spinal Cord Stimulation: Toward Paresthesia-Free Pain Suppression	221	Neuromodulation	De Ridder, Dirk (2010)	Belgium
10	Sustained Effectiveness of 10 kHz High-Frequency Spinal Cord Stimulation for Patients with Chronic, Low Back Pain: 24-Month Results of a Prospective Multicenter Study	218	Pain medicine	Al-Kaisy, Adnan (2014)	England
Ranking	Co-cited literature	Co-cited counts	Journal	First author (year)	Country/Region
1	Spinal Cord Stimulation Versus Conventional Medical Management For Neuropathic Pain: A Multicentre Randomised Controlled Trial In Patients With Failed Back Surgery Syndrome	311	Pain	Kumar, Krishna (2007)	Canada
2	Pain Mechanisms: A New Theory	287	Science	Melzack R (1965)	Canada
3	Spinal Cord Stimulation Versus Repeated Lumbosacral Spine Surgery For Chronic Pain: A Randomized, Controlled Trial	242	Neurosurgery	North, RB (2005)	United States
4	Novel 10-Khz High-Frequency Therapy (HF10 Therapy) Is Superior To Traditional Low-Frequency Spinal Cord Stimulation For The Treatment Of Chronic Back And Leg Pain	233	Anesthesiology	Kapural, Leonardo (2015)	United States
5	The Effects Of Spinal Cord Stimulation In Neuropathic Pain Are Sustained: A 24-Month Follow-Up Of The Prospective Randomized Controlled Multicenter Trial Of The Effectiveness Of Spinal Cord Stimulation	226	Neurosurgery	Kumar, Krishna (2008)	Canada
6	Safety And Efficacy Of Spinal Cord Stimulation For The Treatment Of Chronic Pain: A 20-Year Literature Review	210	Journal of neurosurgery	Cameron, T (2004)	Canada
7	Electrical Inhibition Of Pain By Stimulation Of The Dorsal Columns: Preliminary Clinical Report.	198	Anesthesia and analgesia	Shealy, CN (1967)	United States
Ranking	Co-cited literature	Co-cited counts	Journal	First author (year)	Country/Region
8	Spinal Cord Stimulation In Treatment Of Chronic Benign Pain: Challenges In Treatment Planning And Present Status, A 22-Year Experience	172	Neurosurgery	Kumar, Krishna (2006)	Canada
9	Spinal Cord Stimulation In Patients With Chronic Reflex Sympathetic Dystrophy	169	New England journal of medicine	Kemler, MA (2000)	Netherlands
10	Spinal-Cord Stimulation For Chronic, Intractable Pain - Experience Over 2 Decades	135	Neurosurgery	NORTH, RB (1993)	United States

The top 25 articles with the strongest citation bursts were illustrated in Figure 5B. The red bars represented the periods of the citation bursts, which indicated a higher citation frequency, while the blue bars indicated fewer citations. The strongest burst of literature was published on ANESTHESIOLOGY by Kapural L in 2015, which focused on the application of high-frequency (HF) electrical stimulation for chronic back and leg pain. The most recent paper being concerned was a prospective RCT which successfully confirmed the safety and superior clinical efficacy of a novel, closed-loop system, reported on LANCET NEUROLOGY by Mekhail, N in 2020.

### Keyword analysis

**Figure 6:**
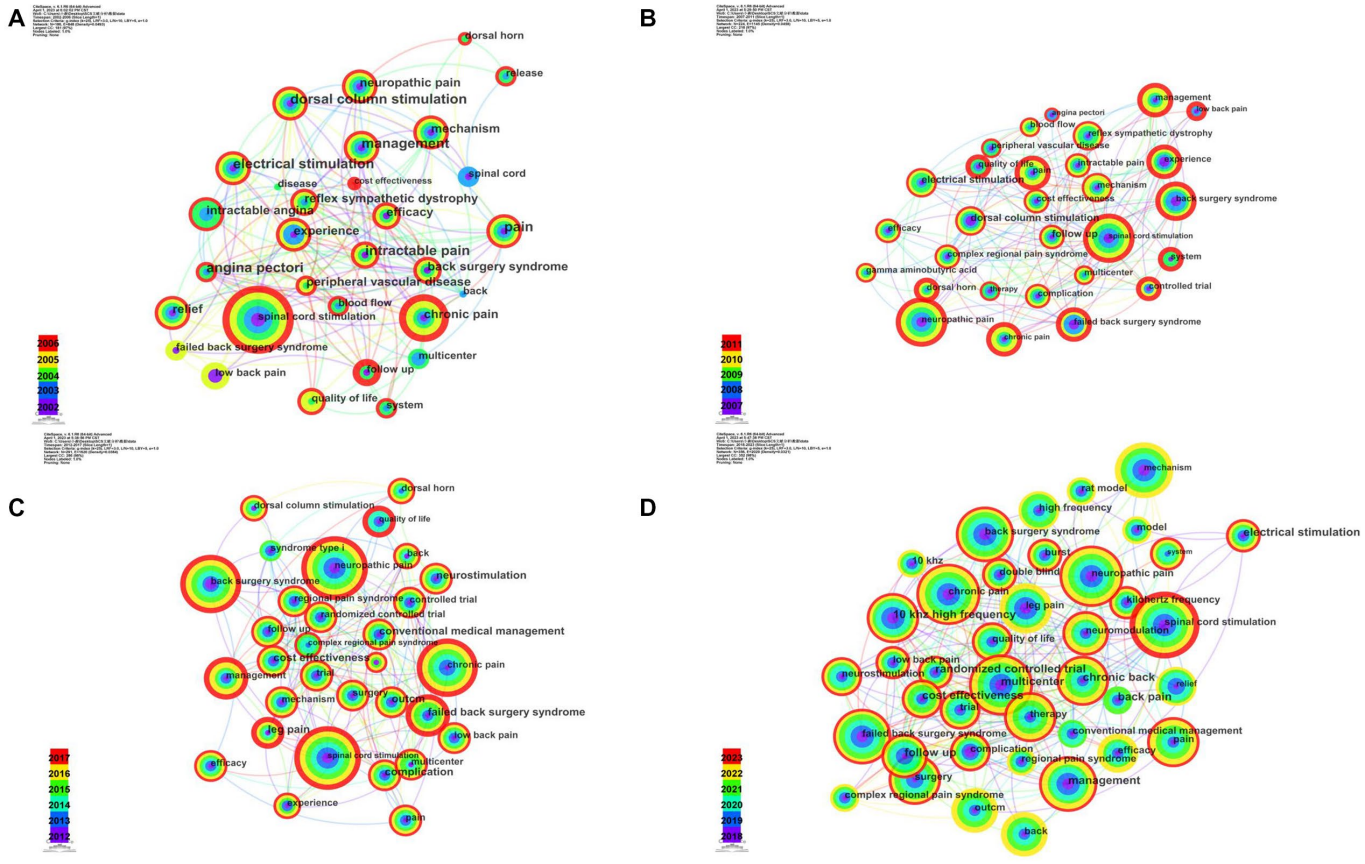
Keyword co-occurrence in five-yearly analysis by CiteSpace. **(A)** The co-occurrence of keywords in 2002–2006. **(B)** The co-occurrence of keywords in 2007–2011. **(C)** The co-occurrence of keywords in 2012–2017. **(D)** The co-occurrence of keywords in 2018–2023.

The five-yearly keyword co-occurrence analysis was presented in Figure 6. The bigger the node was, the more frequently a keyword occurred. The top 10 keywords in each time period and their centralities were summarized in Table 5. Keywords with high research interest were “spinal cord stimulation,” “neuropathic pain,” “chronic pain,” “failed back surgery syndrome (FBSS),” and “management,” etc.

**Table 5:** The top 10 co-occurrence keywords and their centralities in five-yearly analysis.

Ranking	2002–2006			2007–2011		
	Keyword	Frequency	Centrality	Keyword	Frequency	Centrality
1	Spinal cord stimulation	92	0.91	Spinal cord stimulation	180	0.82
2	Chronic pain	36	0.15	Neuropathic pain	71	0.21
3	Electrical stimulation	27	0.13	Back surgery syndrome	43	0.04
4	Neuropathic pain	22	0.13	Chronic pain	42	0.06
5	Pain	21	0.08	pain	37	0.08
6	Management	21	0.07	Management	35	0.08
7	Experience	20	0.05	Experience	32	0.03
8	Dorsal column stimulation	20	0.07	Mechanism	32	0.1
9	Mechanism	19	0.06	Electrical stimulation	27	0.06
10	Back surgery syndrome	18	0.03	Dorsal column Stimulation	23	0.04
Ranking	2012–2017			2018–2023		
	Keyword	Frequency	Centrality	Keyword	Frequency	Centrality
1	Spinal cord stimulation	271	0.61	Spinal cord stimulation	440	0.56
2	Neuropathic pain	155	0.34	Neuropathic pain	179	0.22
3	Chronic pain	97	0.11	Chronic pain	173	0.13
4	Back surgery syndrome	90	0.13	Multicenter	102	0.04
5	Failed back surgery syndrome	61	0.05	Management	92	0.07
6	Management	50	0.04	Pain	88	0.05
7	Pain	45	0.05	10 khz high frequency	81	0.03
8	Randomized controlled trial	44	0.03	Failed back surgery syndrome	78	0.04
9	Follow up	41	0.03	Mechanism	76	0.06
10	Mechanism	37	0.04	Back surgery syndrome	76	0.05

**Figure 7:**
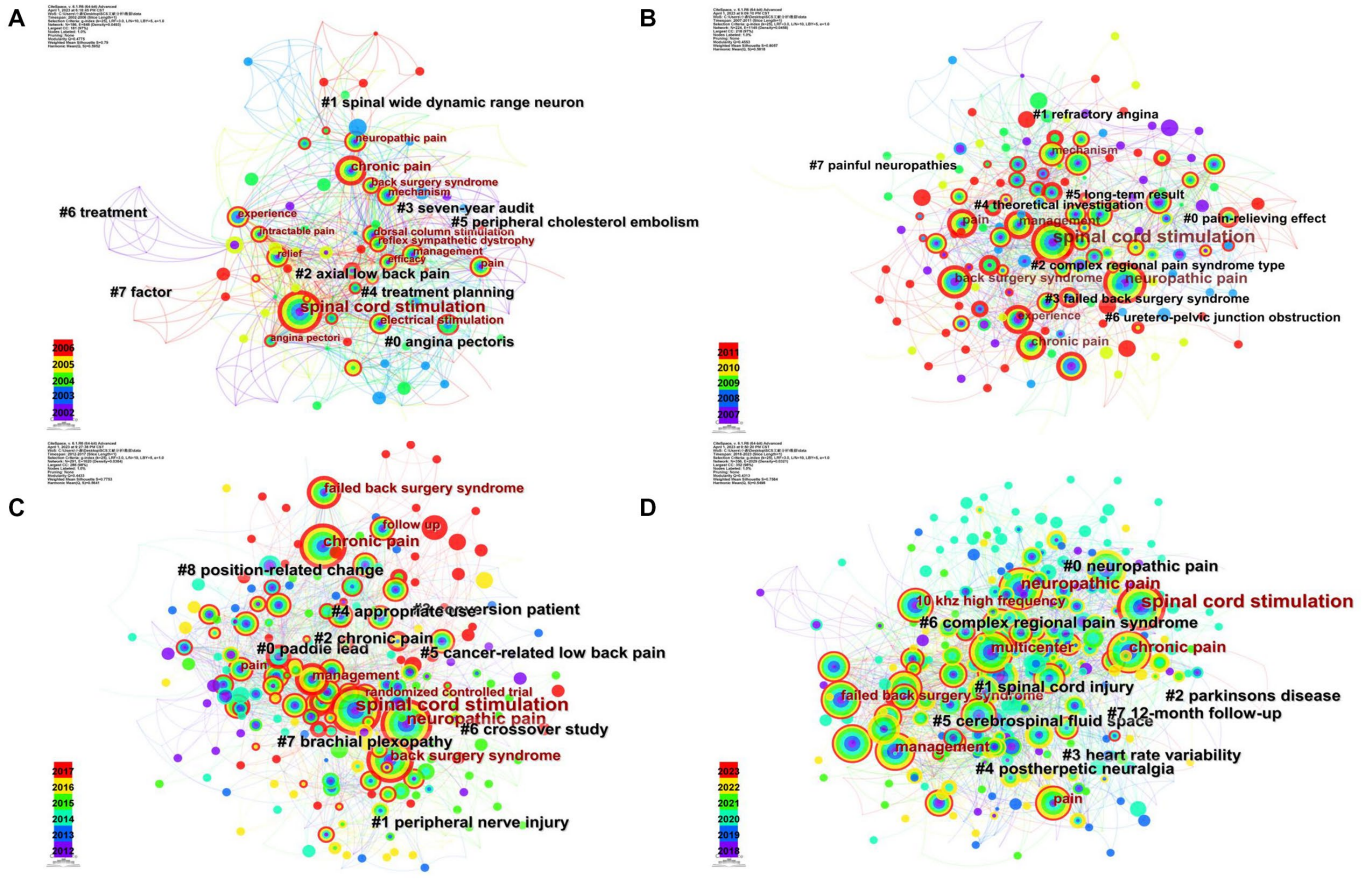
Keyword cluster in five-yearly analysis by CiteSpace. **(A)** The cluster network of keywords in 2002–2006. **(B)** The cluster network of keywords in 2007–2011. **(C)** The cluster network of keywords in 2012–2017. **(D)** The cluster network of keywords in 2018–2023.

Figure 7 showed the cluster analysis of keywords in 5-year intervals. The 2002–2006 keyword clusters were “angina pectoris,” “spinal wide dynamic range neuron,” and “axial low back pain,” etc. “Pain-relieving effect,” “refractory angina,” and “complex regional pain syndrome (CRPS) type,” etc. were the clusters in 2007–2011. Clusters in 2012–2017 were “paddle lead,” “peripheral nerve injury,” and “chronic pain,” etc. While “neuropathic pain,” “spinal cord injury,” and “Parkinson’s disease,” etc. were clusters in 2018–2023.

### Research topics and hotspot trends

**Figure 8:**
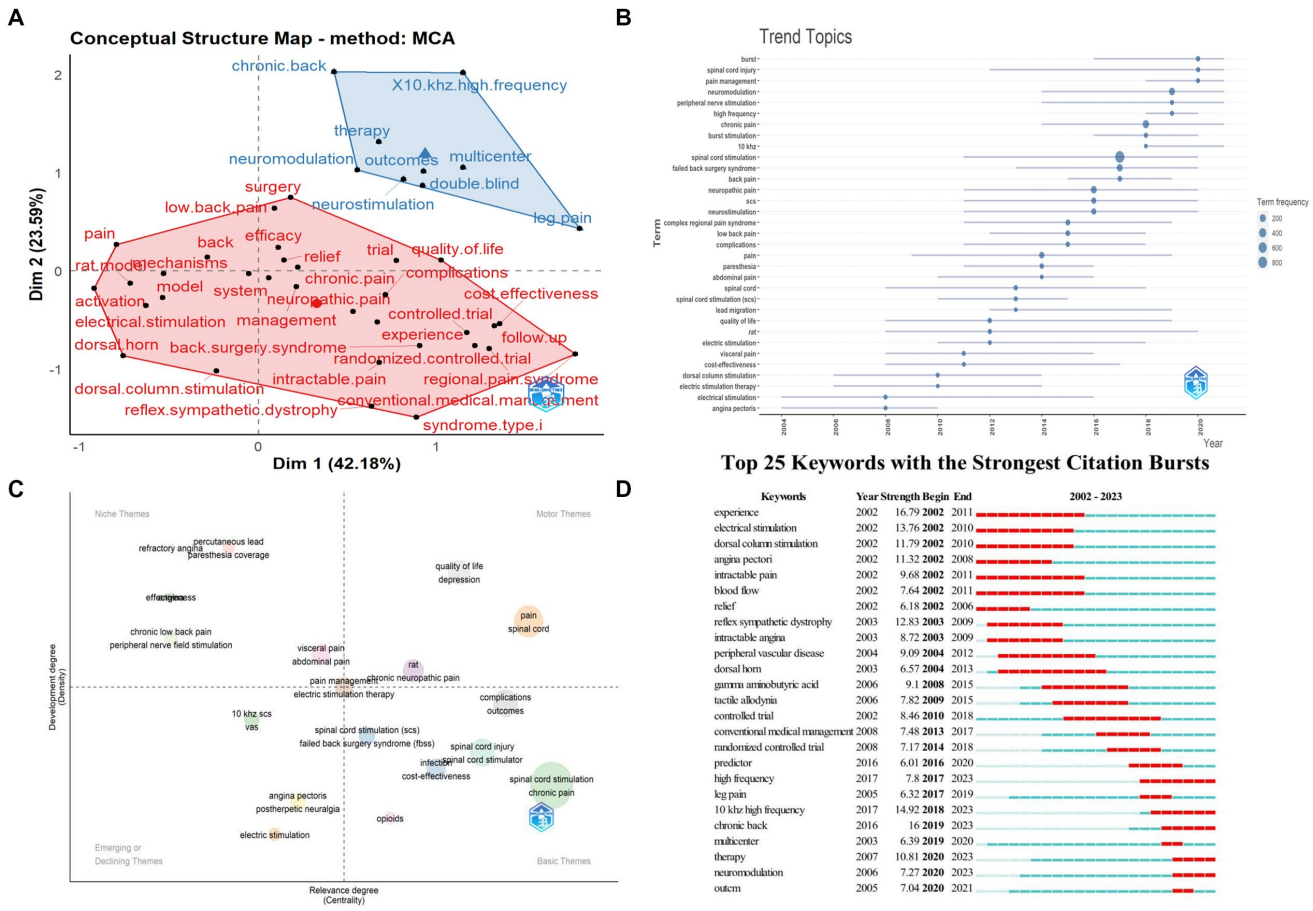
The analysis of keywords. **(A)** Factorial analysis of keywords constructed using multiple correspondence analysis (MCA) and hierarchical clustering techniques by R bibliometrix package. **(B)** Trend topics of keywords by R bibliometrix package. **(C)** Thematic map of keywords by R bibliometrix package. **(D)** The top 25 keywords with the strongest citation bursts by CiteSpace.

The keyword factorial analysis was shown in Figure 8A, which divided the keywords into two clusters. The main keywords in the red cluster were “neuropathic pain” and “low back pain,” indicating those refractory pain. The blue cluster represented therapies and research methods such as “10 khz high frequency,” “multicenter,” and “double blind.”

Global research trends evolution was showed in Figure 8B, the starting segment of the blue line was the time when the keyword first appeared, and the size of the circle was positively related to the number of times that the keyword had appeared. Recent themes were “10khz,” “high frequency,” and “pain management,” etc.

The keywords were divided into four quadrants in the thematic map, as shown in Figure 8C. Themes in the first quadrant were important and well-developed which were represented by “quality of life” and “chronic neuropathic pain.” Themes in the second quadrant had been well-developed but had little impact on the current field, such as “chronic low back pain” and “abdominal pain.” The third quadrant represented marginal themes, such as the “postherpetic neuralgia” and “vas,” which were not so important. The fourth quadrant represented those important but not well-developed themes, mainly referring to basic concepts such as “cost-effectiveness,” “spinal cord stimulation,” and “spinal cord stimulator.”

The citation burst analysis was presented in Figure 8D, the strongest citation burst of keywords was “experience,” followed by “chronic pain,” and “10 khz high frequency,” etc. The most recent keywords being concerned were “neuromodulation,” “therapy” and “outcome,” both starting in 2020.

### Comparison between the United States and Europe

**Figure 9:**
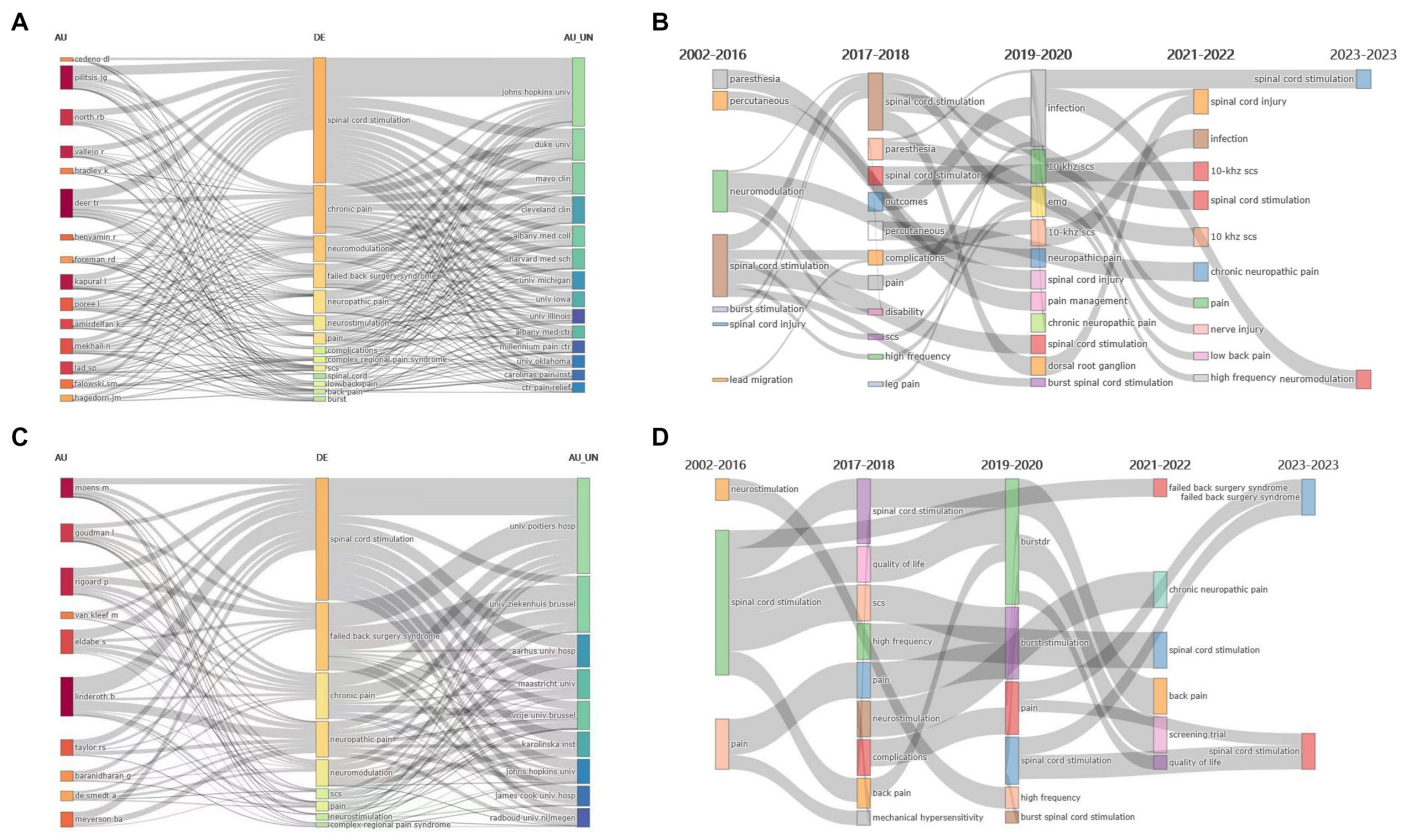
Comparison between the United States and Europe. **(A)** The three-field plot (authors-keywords-institutions) of literature published by the USA. **(B)** The evolution of themes of literature published by the United States. **(C)** The three-field plot (authors-keywords-institutions) of literature published by the Europe. **(D)** The evolution of themes of literature published by Europe.

From the three-field plot analysis (Figures 9A,C), research keywords and institutions or authors were linked by grey lines. We found that the representative authors of the United States were Kapural L, Deer TR, and North RB, their main research themes were “spinal cord stimulation,” “chronic pain,” and “neuromodulation,” etc. The representative institutions of the United States were Johns Hopkins University, Duke University, and Cleveland Clinical, etc. Similarly, Linderoth B, Rigoard P, Eldabe s were the representative authors of the Europe, their main research themes were “spinal cord stimulation,” “failed back surgery syndrome,” and “chronic pain,” etc. The representative institutions of the Europe were Poitiers University Hospital, Universitair Ziekenhuis Brussel, and Aarhus university, etc. The evolution of research themes in the United States and Europe were showed in Figures 9B,D.

## Discussion

Using mapping knowledge domains, we retrospectively analyzed the literature on SCS for pain treatment in the last two decades. As the clinical use of SCS has expanded, researchers are getting more and more attentions to SCS. This study has provided detailed information on advances in SCS treatment for pain management, the leading national and institutional authorities, the publication directions in the field, the representative key findings in this direction, as well as the prediction of future research hotspots.

From 2002 to 2022, researches on SCS for pain management have increased a lot, and the number of citations has increased from 7 to 3,935, reflecting a clear increase in researcher interest. A significant increase emerged in the literature from 2005 to 2009. Combining with co-citation and citation burst analysis of the references, we analyzed that this trend was presumed by the multicenter RCT by Kumar et al. (2007), the largest trial of SCS for neuropathic pain, which achieved over 50% pain relief, significantly exceeding the 30% licensing threshold recommended by the European Medicines Evaluation Agency (EMEA) (Kumar et al., 2007). Because of this exciting result, researchers were encouraged to look to SCS for pain management; what’s more, a review by Cameron (2004), which indicated that SCS was safe and had a long-term effect in the treatment of a wide range of chronic pain, was cited in an upsurge of citations between 2006 and 2009. This had also increased the interest of researchers. Another growth spurt which appeared in 2018, combined with the most popular keyword “10 kHz high frequency” since 2018, might be explained by the 24-month RCT conducted by Kapural et al. (2016). The study of Kapural et al. (2016) sparked research interest in topics such as HF electrical stimulation by confirming that HF stimulation has long-term advantages over conventional low-frequency SCS for chronic back and leg pain. This was also evidenced by the approval from the US Food and Drug Administration (FDA) for 10-kHz HF therapy in the treatment of back and leg pain in 2015 (Kapural et al., 2016). Based on the current positive therapeutic results, it is expected that more attention will be paid to this field in the future, and the emergence of new technologies and application scenarios will contribute to a higher number of publications and total citations.

Regarding the distribution of countries and institutions, United States leads the world leader in the number of publications (749) on SCS for pain treatment, accounting for 54% of the total number of publications and receiving 15,744 citations. This indicated the United States dominance in the field of SCS for pain treatment. In terms of publication number, other countries showed a correlation with their economic levels. For example, Netherlands, England, and Belgium had a higher number of articles published on SCS for pain treatment than other countries. The top five countries in terms of citations were the USA, England, Belgium, Netherlands, and Canada, where SCS research in this field had been widely recognized by researchers. The institution with the highest number of publications in the field of SCS for pain treatment was Johns Hopkins University. The university had conducted extensive research on RCTs and basic physiological experiments in the field of SCS for pain (North et al., 2007; Shechter et al., 2013; Yang et al., 2015; Su et al., 2017; Deer et al., 2018), covering various applications of SCS for chronic low back pain (North et al., 2005), angina (Rea and Erdek, 2007), cancer pain (Soffin et al., 2012), neuropathic pain (Dworkin et al., 2013), and other pain conditions. Other prominent institutions with significant contributions to research on SCS for pain treatment included Albany Medical Center, the Karolinska Institute, and Cleveland Clinic. In terms of citations, Johns Hopkins University, the Karolinska Institute, Cleveland Clinic, Nevro Corporation, Duke University, and Albany Medical Center had been widely recognized for their research. These institutions are available to researchers for studying, exchanging, and collaborating.

The quality of research in this field was generally high, with 50% of SCS research published in journals with a Q2 division or higher, and a minimum impact factor of over three. The journal with the highest number of publications in this field was NEUROMODULATION, which focused mainly on clinical, translational, and basic research on neuromodulation (Brandmeir and Sather, 2015; Duan et al., 2021). There are some other journals with high impact factors, such as PAIN, PAIN PHYSICIAN, and NEUROSURGERY. These journals publish a significant number of papers with high co-citations on SCS for pain treatment, making them valuable resources for researchers to study.

The authors who had made a significant contribution to the field of SCS for pain treatment are Linderoth B, Deer TR, and Pilitsis JG. Professor Linderoth B, from the Karolinska Institutet, is working on the mechanism of SCS in pain relief, such as the increased 5-hydroxytryptamine (5-HT) release, the inhibition of excitatory amino acids (EAA) release by increased gamma-aminobutyric acid (GABA) release, and the activation of the cholinergic system in the dorsal horn in SCS pain control (Linderoth et al., 1994; Stiller et al., 1996; Schechtmann et al., 2008; Song et al., 2011). Professor Deer TR from Spine & Nerve Center, University of West Virginia, is an expert who focuses on intrathecal drug delivery and neuromodulation of pain control, especially in dorsal root ganglion stimulation (DRGS), SCS, and peripheral nerve stimulation (PNS) (Deer et al., 2011, 2016, 2019; Mekhail et al., 2020). Professor Pilitsis JG from Florida Atlantic University, has finished many SCS clinical trials, she dedicated to the research of prognosis and pain assessment in patients that treated with SCS as well, which all acquired satisfied outcomes (Youn et al., 2015; Marola et al., 2017; Paul et al., 2017; Kumar et al., 2018; Gee et al., 2019). These findings can be useful for basic and clinical researchers who are seeking the literature for their clinical studies or basic research.

The co-citation analysis of authors and literature, along with keyword co-occurrence and hierarchical clustering by multiple correspondence analysis (MCA), highlighted hot topics in the research field and how they have evolved over time (Chen, 2017). The three most highly co-cited authors were Kumar K, Deer TR, and Kapural L. As a neurosurgeon, Kumar K had conducted many clinical studies on SCS, covering a wide range of areas, including applications, management of complications, and cost–benefit analysis (Kumar et al., 2002, 2005; Deer et al., 2014). Deer TR, the most published author in this field, had also received a large number of co-citations. Kapural L completed many RCT and clinical trials, demonstrated the effectiveness and safety of SCS in the treatment of chronic back and leg pain (Kapural et al., 2016, 2017; Amirdelfan et al., 2018; Levy et al., 2019, 2020; Mekhail et al., 2020). Meanwhile, he introduced SCS into the treatment of chronic abdominal pain and achieved satisfactory results (Kapural and Rakic, 2008; Puylaert et al., 2011; Kapural et al., 2020). The most highly co-cited paper in our analysis was an RCT by Professor Kumar K on the effects of SCS and conventional medication in patients with FBSS, which showed that the analgesic effect of SCS was more satisfactory (Kumar et al., 2007). The second most highly co-cited paper was Melzack R’s 1965 gate-control theory of pain transmission, which provided the physiological basis for SCS (Melzack and Wall, 1965). The report on the first clinical application of SCS for pain treatment in 1967 also received a significant number of co-citations. The keyword co-occurrence analysis at 5-year intervals (Figure 6) showed that SCS was initially used for neuropathic pain and chronic pain, with a focus on chronic pain being mainly low back pain, and the keyword “mechanism” appeared more frequently in each time period. In summary, since its first clinical use on pain treatment in 1967, SCS has been the subject of numerous studies that have confirmed its effectiveness and benefits in various clinical settings. As shown in the hierarchical cluster of factorial analysis (Figure 8A), the current research on SCS mainly consists of two aspects. The first is various types of refractory pain, represented by neuropathic pain and FBSS, and the second is therapy and research methods, etc., indicating that SCS has been continuously expanded in clinical pain application scenarios by multicenter research since its birth. We can learn that FBSS and clinical trial or RCT of SCS on various pain scenarios are hotspots in the field. Meanwhile, the mechanism of SCS for pain control is also attractive and important, maybe we can improve the SCS based on a clearly defined physiological mechanism in the future.

The most recent research trends in the field were revealed by a burst analysis of literature and keywords, as well as the thematic map of keywords. The paper with the strongest citation burst was published on ANESTHESIOLOGY by Kapural L et al. in 2015. The study highlighted the effectiveness of HF electrical stimulation, with results showing that this new HF therapy was almost twice as effective as conventional therapy in the long-term treatment of back and leg pain (Kapural et al., 2015). The paper that had received growing attention latest was Deer TR et al.’s study on burst waveforms, also as a new technique for SCS, and it revealed that burst waveforms were more effective in treating chronic pain than conventional SCS (Deer et al., 2018). However, what is being done with the SCS technique has attracted the attention of researchers. The study by Deer TR et al. stated that DRGS was more effective than SCS for pain control in patients with CRPS type I and causalgia (Deer et al., 2017). A systematic review by Grider J et al. had gotten the same attention, which assessed the effectiveness of SCS for chronic spinal pain and concluded that there was significant evidence (grades I–II) for its efficacy of SCS for lumbar FBSS (Grider et al., 2016). In addition, the highest citation burst keyword was “experience,” and other keywords that had received attention in the last 5 years included “10 khz high frequency,” “therapy,” “multicenter,” “chronic back,” “therapy,” and “outcome.” These findings revealed that the most recent research has shifted towards the use of HF electrical stimulation, and multicenter trials are often used to validate the findings in this area. Moreover, as shown in the thematic map of keywords (Figure 8C), research of SCS in “chronic low back pain” and “refractory angina” had been relatively mature (the second quadrant), with a trend of gradually turning to the field of “chronic neuropathic pain” (the first quadrant), which already had a certain research basis. As for the basic concepts of SCS on pain treatment, such as “cost-effectiveness,” “spinal cord stimulator,” and “complications” (the fourth quadrant), the current research is still immature, and many issues have not been fully elucidated. Combined with the analysis of trend topics, 10 khz-HF SCS (De Carolis et al., 2017; Kapural et al., 2020; Gupta et al., 2021; Kapural and Calodney, 2022; Hasoon et al., 2023), burst SCS (De Ridder et al., 2010, 2013; Schu et al., 2014; Deer et al., 2018), SCS combined application with PNS (Rigoard et al., 2021), HF-SCS (Song et al., 2015; Kowalski et al., 2016, 2017), close loop (Mekhail et al., 2020, 2022), adapter (Rigoard et al., 2022), predictive responders (Sparkes et al., 2015; Goudman et al., 2021; Ounajim et al., 2021), combined waveforms (Billot et al., 2020; Kallewaard et al., 2021), and holistic/multidimensional assessment (Pilitsis et al., 2021; Rigoard et al., 2021; Goudman et al., 2023; Levy et al., 2023) would be the future trends in pain management. In conclusion, we predict that applications in more types of pain, study of complications, development and optimization of SCS techniques, and combined application of SCS with other neuromodulation to treat pain would be the future research trends.

Additionally, in terms of SCS research, both American and European researchers have used SCS for the treatment of a variety types of pain as well as conducting many studies on complications and infections after SCS implantation. However, European researchers showed more interest in burst stimulation (De Ridder et al., 2010, 2013; Schu et al., 2014), FBSS pain control (Zucco et al., 2015), the research of complications, risk of infection, and quality of life after implantation of SCS (Kumar et al., 2006, 2007; Deer et al., 2014), while American researchers were more interested in developing new technologies such as HF stimulation (De Carolis et al., 2017; Kissoon et al., 2017; Kapural et al., 2020; Gupta et al., 2021; Kapural and Calodney, 2022).

Spinal cord stimulation has been used clinically for over 50 years for pain treatment, and has been proven to be effective in clinical trials. In the last two decades, SCS has developed rapidly, with new techniques such as HF electrical stimulation being developed and tested (Kapural et al., 2015), and United States has been a leading country in this regard. SCS clinical trials have gradually become a research hotspot, and the proportion of basic research has gradually decreased. It is vital to remark that the development of new technologies and their applications are hot research topics.

It is important to note that the study has a few limitations. First, the analysis was based on the literature from the last two decades and might have slightly affected the results of the analysis of developments. Second, based on WOSCC and English language literature, this study might have excluded a few important articles that were written in other languages. Finally, the more streamlined search format might have included a small number of articles that were not directly related to the field; however, this would not significantly impact the overall understanding of our study.

Overall, our study provides a comprehensive view of the development of pain control in SCS over the last two decades, enabling future researchers to identify the most prominent countries, institutions, and authors, etc. in the field more quickly. At the same time, we not only identified many highly regarded literature and keywords in the field but also evaluated their corresponding temporal information, and scientifically predicted the research hotspots and trends.

## Data availability statement

The raw data supporting the conclusions of this article will be made available by the authors, without undue reservation.

## Author contributions

SH, XL, and GF designed the study and critically revised the manuscript. SY was responsible for project implementation and administration. SZ performed the statistical analyses. YF extracted the data and performed the data preprocessing. YZ wrote the original draft. NX and YL performed the data visualization and results interpretation. All authors reviewed and edited the final manuscript for submission.

## Funding

This work was funded by the Science and Technology Commission of Shanghai Municipality (Grant No. 22S31900100), Yunnan Academician Expert Workstation (Grant No. 202205AF150058), the National Natural Science Foundation of China (Grant No. 82102640), the Medical Scientific Research Foundation of Guangdong Province of China (Grant No. A202319), and the National Key Research and Development Program of China (Grant No. 2022YFC3602203). The funders had no role in study design, data collection, data analysis, interpretation, writing of this report and in the decision to submit the paper for publication.

## Conflict of interest

The authors declare that the research was conducted in the absence of any commercial or financial relationships that could be construed as a potential conflict of interest.

## Publisher’s note

All claims expressed in this article are solely those of the authors and do not necessarily represent those of their affiliated organizations, or those of the publisher, the editors and the reviewers. Any product that may be evaluated in this article, or claim that may be made by its manufacturer, is not guaranteed or endorsed by the publisher.

## Abbreviations

SCS: spinal cord stimulation
WOSCC: Web of Science Core Collection
RCT: randomized controlled trial
HF: high-frequency
FBSS: failed back surgery syndrome
CRPS: complex regional pain syndrome
EMEA: European Medicines Evaluation Agency
FDA: Food and Drug Administration
DRGS: dorsal root ganglion stimulation
PNS: peripheral nerve stimulation
5-HT: 5-hydroxytryptamine
EAA: excitatory amino acids
GABA: gamma-aminobutyric acid
MCA: multiple correspondence analysis

## References

[ref1] Amirdelfan K. Yu C. Doust M. W. Gliner B. E. Morgan D. M. Kapural L.. (2018). Long-term quality of life improvement for chronic intractable back and leg pain patients using spinal cord stimulation: 12-month results from the SENZA-RCT. Qual. Life Res. 27, 2035–2044. doi: 10.1007/s11136-018-1890-8, PMID: 29858746

[ref2] Billot M. Daycard M. Wood C. Tchalla A. (2019). Reiki therapy for pain, anxiety and quality of life. BMJ Support. Palliat. Care 9, bmjspcare-2019-001775–bmjspcare-2019-001778. doi: 10.1136/bmjspcare-2019-001775, PMID: 30948444

[ref3] Billot M. Naiditch N. Brandet C. Lorgeoux B. Baron S. Ounajim A.. (2020). Comparison of conventional, burst and high-frequency spinal cord stimulation on pain relief in refractory failed back surgery syndrome patients: study protocol for a prospective randomized double-blinded cross-over trial (MULTIWAVE study). Trials 21:696. doi: 10.1186/s13063-020-04587-6, PMID: 32746899 PMC7397663

[ref4] Brandmeir N. J. Sather M. D. (2015). Spinal cord stimulation for the treatment of neuropathic pain associated with leprosy: a case report. Neuromodulation 18, 762–764. doi: 10.1111/ner.12295, PMID: 25864772

[ref5] Breivik H. Eisenberg E. O’Brien T. (2013). The individual and societal burden of chronic pain in Europe: the case for strategic prioritisation and action to improve knowledge and availability of appropriate care. BMC Public Health 13:1229. doi: 10.1186/1471-2458-13-1229, PMID: 24365383 PMC3878786

[ref6] Cameron T. (2004). Safety and efficacy of spinal cord stimulation for the treatment of chronic pain: a 20-year literature review. J. Neurosurg. 100, 254–267. doi: 10.3171/spi.2004.100.3.0254, PMID: 15029914

[ref7] Chen C. M. (2017). Science mapping: a systematic review of the literature. J Data Info Sci 2, 1–40. doi: 10.1515/jdis-2017-0006

[ref8] Cohen S. P. Vase L. Hooten W. M. (2021). Chronic pain: an update on burden, best practices, and new advances. Lancet 397, 2082–2097. doi: 10.1016/s0140-6736(21)00393-7, PMID: 34062143

[ref9] De Carolis G. Paroli M. Tollapi L. Doust M. W. Burgher A. H. Yu C.. (2017). Paresthesia-Independence: an assessment of technical factors related to 10 kHz Paresthesia-free spinal cord stimulation. Pain Physician 20, 331–341. doi: 10.36076/ppj.2017.341, PMID: 28535555

[ref10] De Ridder D. Plazier M. Kamerling N. Menovsky T. Vanneste S. (2013). Burst spinal cord stimulation for limb and back pain. World Neurosurg. 80, 642–9.e1. doi: 10.1016/j.wneu.2013.01.040, PMID: 23321375

[ref11] De Ridder D. Vanneste S. Plazier M. van der Loo E. Menovsky T. (2010). Burst spinal cord stimulation: toward paresthesia-free pain suppression. Neurosurgery 66, 986–990. doi: 10.1227/01.Neu.0000368153.44883.B3 20404705

[ref12] Deer T. R. Levy R. M. Kramer J. Poree L. Amirdelfan K. Grigsby E.. (2017). Dorsal root ganglion stimulation yielded higher treatment success rate for complex regional pain syndrome and causalgia at 3 and 12 months: a randomized comparative trial. Pain 158, 669–681. doi: 10.1097/j.pain.0000000000000814, PMID: 28030470 PMC5359787

[ref13] Deer T. R. Mekhail N. Provenzano D. Pope J. Krames E. Thomson S.. (2014). The appropriate use of neurostimulation: avoidance and treatment of complications of neurostimulation therapies for the treatment of chronic pain. Neuromodulation appropriateness consensus committee. Neuromodulation 17, 571–597. doi: 10.1111/ner.12206, PMID: 25112891

[ref14] Deer T. Pope J. Benyamin R. Vallejo R. Friedman A. Caraway D.. (2016). Prospective, multicenter, randomized, double-blinded, partial crossover study to assess the safety and efficacy of the novel neuromodulation system in the treatment of patients with chronic pain of peripheral nerve origin. Neuromodulation 19, 91–100. doi: 10.1111/ner.12381, PMID: 26799373

[ref15] Deer T. R. Pope J. E. Lamer T. J. Grider J. S. Provenzano D. Lubenow T. R.. (2019). The Neuromodulation appropriateness consensus committee on best practices for dorsal root ganglion stimulation. Neuromodulation 22, 1–35. doi: 10.1111/ner.12845, PMID: 30246899

[ref16] Deer T. Slavin K. V. Amirdelfan K. North R. B. Burton A. W. Yearwood T. L.. (2018). Success using Neuromodulation with BURST (SUNBURST) study: results from a prospective, randomized controlled trial using a novel burst waveform. Neuromodulation 21, 56–66. doi: 10.1111/ner.12698, PMID: 28961366

[ref17] Deer T. R. Smith H. S. Burton A. W. Pope J. E. Doleys D. M. Levy R. M.. (2011). Comprehensive consensus based guidelines on Intrathecal drug delivery systems in the treatment of pain caused by cancer pain. Pain Physician 14, E283–E312. doi: 10.36076/ppj.2011/14/E283, PMID: 21587338

[ref18] Duan W. R. Huang Q. Yang F. He S. Q. Guan Y. (2021). Spinal cord stimulation attenuates below-level mechanical hypersensitivity in rats after thoracic spinal cord injury. Neuromodulation 24, 33–42. doi: 10.1111/ner.13248, PMID: 32770848 PMC7855640

[ref19] Dworkin R. H. O’Connor A. B. Kent J. Mackey S. C. Raja S. N. Stacey B. R.. (2013). Interventional management of neuropathic pain: NeuPSIG recommendations. Pain 154, 2249–2261. doi: 10.1016/j.pain.2013.06.004, PMID: 23748119 PMC4484720

[ref20] Furlan A. D. Giraldo M. Baskwill A. Irvin E. Imamura M. (2015). Massage for low-back pain. Cochrane Database Syst. Rev. 2015:CD001929. doi: 10.1002/14651858.CD001929.pub3, PMID: 26329399 PMC8734598

[ref21] Garza-Villarreal E. A. Pando V. Vuust P. Parsons C. (2017). Music-induced Analgesia in chronic pain conditions: a systematic review and meta-analysis. Pain Physician 20, 597–610. doi: 10.36076/ppj/2017.7.597, PMID: 29149141

[ref22] Gaskin D. J. Richard P. (2012). The economic costs of pain in the United States. J. Pain 13, 715–724. doi: 10.1016/j.jpain.2012.03.009 22607834

[ref23] Gee L. Smith H. C. Ghulam-Jelani Z. Khan H. Prusik J. Feustel P. J.. (2019). Spinal cord stimulation for the treatment of chronic pain reduces opioid use and results in superior clinical outcomes when used without opioids. Neurosurgery 84, 217–226. doi: 10.1093/neuros/nyy065, PMID: 29538696

[ref24] Goudman L. Billot M. Duarte R. V. Eldabe S. Rigoard P. Moens M. (2023). Gradation of clinical holistic response as new composite outcome to evaluate success in spinal cord stimulation studies for pain. Neuromodulation 26, 139–146. doi: 10.1016/j.neurom.2021.10.020, PMID: 35088757

[ref25] Goudman L. De Smedt A. Eldabe S. Rigoard P. Linderoth B. De Jaeger M.. (2021). High-dose spinal cord stimulation for patients with failed back surgery syndrome: a multicenter effectiveness and prediction study. Pain 162, 582–590. doi: 10.1097/j.pain.0000000000002035, PMID: 32910099

[ref26] Goudman L. Putman K. Van Doorslaer L. Billot M. Roulaud M. Rigoard P.. (2023). Proportion of clinical holistic responders in patients with persistent spinal pain syndrome type II treated by subthreshold spinal cord stimulation compared to best medical treatment: a study protocol for a multicentric randomised controlled trial (TRADITION). Trials 24:120. doi: 10.1186/s13063-023-07140-3, PMID: 36803412 PMC9940414

[ref27] Grider J. S. Manchikanti L. Carayannopoulos A. Sharma M. L. Balog C. C. Harned M. E.. (2016). Effectiveness of spinal cord stimulation in chronic spinal pain: a systematic review. Pain Physician 19, E33–E54. doi: 10.36076/ppj/2016.19.E33, PMID: 26752493

[ref28] Gupta M. Ray M. Ladesich N. Gupta A. (2021). Health-care utilization and outcomes with 10 kHz spinal cord stimulation for chronic refractory pain. J. Pain Res. 14, 3675–3683. doi: 10.2147/jpr.S306126, PMID: 34880672 PMC8648088

[ref29] Hasoon J. Robinson C. Urits I. Viswanath O. Kaye A. D. (2023). Utilizing 10kHz stimulation to salvage a failed low frequency spinal cord stimulation trial. Orthop. Rev. (Pavia) 15:57624. doi: 10.52965/001c.57624, PMID: 36776275 PMC9907322

[ref30] Hilton L. Hempel S. Ewing B. A. Apaydin E. Xenakis L. Newberry S.. (2017). Mindfulness meditation for chronic pain: systematic review and meta-analysis. Ann. Behav. Med. 51, 199–213. doi: 10.1007/s12160-016-9844-2, PMID: 27658913 PMC5368208

[ref31] Kallewaard J. W. Paz-Solis J. F. De Negri P. Canós-Verdecho M. A. Belaid H. Thomson S. J.. (2021). Real-world outcomes using a spinal cord stimulation device capable of combination therapy for chronic pain: a European, multicenter experience. J. Clin. Med. 10:11. doi: 10.3390/jcm10184085, PMID: 34575196 PMC8466217

[ref32] Kapural L. Calodney A. (2022). Retrospective efficacy and cost-containment assessment of 10 kHz spinal cord stimulation (SCS) in non-surgical refractory Back pain patients. J. Pain Res. 15, 3589–3595. doi: 10.2147/jpr.S373873, PMID: 36415659 PMC9676005

[ref33] Kapural L. Gupta M. Paicius R. M. Strodtbeck W. Vorenkamp K. E. Gilmore C.. (2020). Treatment of chronic abdominal pain with 10-kHz spinal cord stimulation: safety and efficacy results from a 12-month prospective, multicenter, feasibility study. Clin. Transl. Gastroenterol. 11:10. doi: 10.14309/ctg.0000000000000133, PMID: 32463618 PMC7145032

[ref34] Kapural L. Peterson E. Provenzano D. A. Staats P. (2017). Clinical evidence for spinal cord stimulation for failed back surgery syndrome (FBSS). Spine 42, S61–S66. doi: 10.1097/brs.0000000000002213, PMID: 28441313

[ref35] Kapural L. Rakic M. (2008). Spinal cord stimulation for chronic visceral pain secondary to chronic non-alcoholic pancreatitis. J. Clin. Gastroenterol. 42, 750–751. doi: 10.1097/01.mcg.0000225647.77437.45, PMID: 18496389

[ref36] Kapural L. Sayed D. Kim B. Harstroem C. Deering J. (2020). Retrospective assessment of salvage to 10 kHz spinal cord stimulation (SCS) in patients who failed traditional SCS therapy: RESCUE study. J. Pain Res. 13, 2861–2867. doi: 10.2147/jpr.S281749, PMID: 33204147 PMC7667504

[ref37] Kapural L. Yu C. Doust M. W. Gliner B. E. Vallejo R. Sitzman B. T.. (2015). Novel 10-kHz high-frequency therapy (HF10 therapy) is superior to traditional low-frequency spinal cord stimulation for the treatment of chronic Back and leg pain: the SENZA-RCT randomized controlled trial. Anesthesiology 123, 851–860. doi: 10.1097/aln.0000000000000774, PMID: 26218762

[ref38] Kapural L. Yu C. Doust M. W. Gliner B. E. Vallejo R. Sitzman B. T.. (2016). Comparison of 10-kHz high-frequency and traditional low-frequency spinal cord stimulation for the treatment of chronic back and leg pain: 24-month results from a multicenter, randomized, controlled pivotal trial. Neurosurgery 79, 667–677. doi: 10.1227/neu.0000000000001418, PMID: 27584814 PMC5058646

[ref39] Keathley-Herring H. Van Aken E. Gonzalez-Aleu F. Deschamps F. Letens G. Orlandini P. C. (2016). Assessing the maturity of a research area: bibliometric review and proposed framework. Scientometrics 109, 927–951. doi: 10.1007/s11192-016-2096-x

[ref40] Kissoon N. R. Hoelzer B. C. Martin D. P. Lamer T. J. (2017). High-frequency spinal cord stimulation in a patient with an implanted cardiac device. Pain Pract. 17, 558–563. doi: 10.1111/papr.12530, PMID: 27770599

[ref41] Knotkova H. Hamani C. Sivanesan E. Le Beuffe M. F. E. Moon J. Y. Cohen S. P.. (2021). Neuromodulation for chronic pain. Lancet 397, 2111–2124. doi: 10.1016/s0140-6736(21)00794-7 34062145

[ref42] Kowalski K. E. Romaniuk J. R. Brose S. W. Richmond M. A. Kowalski T. DiMarco A. F. (2016). High frequency spinal cord stimulation-new method to restore cough. Respir. Physiol. Neurobiol. 232, 54–56. doi: 10.1016/j.resp.2016.07.001, PMID: 27395446 PMC5012955

[ref43] Kowalski K. E. Romaniuk J. R. Kowalski T. DiMarco A. F. (2017). Effects of expiratory muscle activation via high-frequency spinal cord stimulation. J. Appl. Physiol. (1985) 123, 1525–1531. doi: 10.1152/japplphysiol.00402.2017 28935824

[ref44] Kumar K. Buchser E. Linderoth B. Meglio M. Van Buyten J. P. (2007). Avoiding complications from spinal cord stimulation: practical recommendations from an international panel of experts. Neuromodulation 10, 24–33. doi: 10.1111/j.1525-1403.2007.00084.x, PMID: 22151809

[ref45] Kumar K. Malik S. Demeria D. (2002). Treatment of chronic pain with spinal cord stimulation versus alternative therapies: cost-effectiveness analysis. Neurosurgery 51, 106–116. doi: 10.1097/00006123-200207000-00016, PMID: 12182407

[ref46] Kumar K. North R. Taylor R. Sculpher M. Van den Abeele C. Gehring M.. (2005). Spinal cord stimulation vs. conventional medical management: a prospective, randomized, controlled, multicenter study of patients with failed back surgery syndrome (PROCESS study). Neuromodulation 8, 213–218. doi: 10.1111/j.1525-1403.2005.00027.x, PMID: 22151547

[ref47] Kumar V. Prusik J. Lin Y. F. Hwang R. Feustel P. Pilitsis J. G. (2018). Efficacy of alternating conventional stimulation and high frequency stimulation in improving spinal cord stimulation outcomes: a pilot study. Neuromodulation 21, 466–471. doi: 10.1111/ner.12755, PMID: 29405548

[ref48] Kumar K. Taylor R. S. Jacques L. Eldabe S. Meglio M. Molet J.. (2007). Spinal cord stimulation versus conventional medical management for neuropathic pain: a multicentre randomised controlled trial in patients with failed back surgery syndrome. Pain 132, 179–188. doi: 10.1016/j.pain.2007.07.028, PMID: 17845835

[ref49] Kumar K. Wilson J. R. Taylor R. S. Gupta S. (2006). Complications of spinal cord stimulation, suggestions to improve outcome, and financial impact. J. Neurosurg. Spine 5, 191–203. doi: 10.3171/spi.2006.5.3.191, PMID: 16961079

[ref50] Levy R. Deer T. R. Poree L. Rosen S. M. Kapural L. Amirdelfan K.. (2019). Multicenter, randomized, double-blind study protocol using human spinal cord recording comparing safety, efficacy, and neurophysiological responses between patients being treated with evoked compound action potential-controlled closed-loop spinal cord stimulation or open-loop spinal cord stimulation (the evoke study). Neuromodulation 22, 317–326. doi: 10.1111/ner.12932, PMID: 30828946

[ref51] Levy R. M. Mekhail N. Abd-Elsayed A. Abejón D. Anitescu M. Deer T. R.. (2023). Holistic treatment response: an international expert panel definition and criteria for a new paradigm in the assessment of clinical outcomes of spinal cord stimulation. Neuromodulation. doi: 10.1016/j.neurom.2022.11.011, PMID: 36604242

[ref52] Levy R. M. Mekhail N. Kramer J. Poree L. Amirdelfan K. Grigsby E.. (2020). Therapy habituation at 12 months: spinal cord stimulation versus dorsal root ganglion stimulation for complex regional pain syndrome type I and II. J. Pain 21, 399–408. doi: 10.1016/j.jpain.2019.08.005, PMID: 31494275

[ref53] Linderoth B. Stiller C. O. Gunasekera L. Oconnor W. T. Ungerstedt U. Brodin E. (1994). Gamma-aminobutyric-acid is released in the dorsal horn by electrical spinal-cord stimulation-an in-vivo microdialysis study in the rat. Neurosurgery 34, 484–489. doi: 10.1227/00006123-199403000-00014 8190224

[ref54] Marola O. Cherala R. Prusik J. Kumar V. Fama C. Wilock M.. (2017). BMI as a predictor of spinal cord stimulation success in chronic pain patients. Neuromodulation 20, 269–273. doi: 10.1111/ner.12482, PMID: 27491832

[ref55] Mekhail N. Levy R. M. Deer T. R. Kapural L. Li S. A. Amirdelfan K.. (2020). Long-term safety and efficacy of closed-loop spinal cord stimulation to treat chronic back and leg pain (Evoke): a double-blind, randomised, controlled trial. Lancet Neurol. 19, 123–134. doi: 10.1016/s1474-4422(19)30414-4, PMID: 31870766

[ref56] Mekhail N. Levy R. M. Deer T. R. Kapural L. Li S. Amirdelfan K.. (2022). Durability of clinical and quality-of-life outcomes of closed-loop spinal cord stimulation for chronic Back and leg pain: a secondary analysis of the Evoke randomized clinical trial. JAMA Neurol. 79, 251–260. doi: 10.1001/jamaneurol.2021.4998, PMID: 34998276 PMC8742908

[ref57] Melzack R. Wall P. D. (1965). Pain mechanisms: a new theory. Science 150, 971–979. doi: 10.1126/science.150.3699.971 5320816

[ref58] Mills S. E. E. Nicolson K. P. Smith B. H. (2019). Chronic pain: a review of its epidemiology and associated factors in population-based studies. Br. J. Anaesth. 123, e273–e283. doi: 10.1016/j.bja.2019.03.023, PMID: 31079836 PMC6676152

[ref59] North R. B. Kidd D. H. Farrokhi F. Piantadosi S. A. (2005). Spinal cord stimulation versus repeated lumbosacral spine surgery for chronic pain: a randomized, controlled trial. Neurosurgery 56, 98–107. doi: 10.1227/01neu.0000144839.65524.E0, PMID: 15617591

[ref60] North R. B. Kidd D. H. Olin J. Sieracki J. M. Boulay M. (2007). Spinal cord stimulation with interleaved pulses: a randomized, controlled trial. Neuromodulation 10, 349–357. doi: 10.1111/j.1525-1403.2007.00123.x, PMID: 22150894

[ref61] Ounajim A. Billot M. Goudman L. Louis P. Y. Slaoui Y. Roulaud M.. (2021). Machine learning algorithms provide greater prediction of response to SCS than Lead screening trial: a predictive AI-based multicenter study. J. Clin. Med. 10:17. doi: 10.3390/jcm10204764, PMID: 34682887 PMC8538165

[ref62] Paley C. A. Johnson M. I. (2020). Acupuncture for the relief of chronic pain: a synthesis of systematic reviews. Medicina 56:6. doi: 10.3390/medicina56010006, PMID: 31878346 PMC7023333

[ref63] Paul A. R. Kumar V. Roth S. Gooch M. R. Pilitsis J. G. (2017). Establishing minimal clinically important difference of spinal cord stimulation therapy in post-laminectomy syndrome. Neurosurgery 81, 1011–1015. doi: 10.1093/neuros/nyx153, PMID: 28973581

[ref64] Pilitsis J. G. Fahey M. Custozzo A. Chakravarthy K. Capobianco R. (2021). Composite score is a better reflection of patient response to chronic pain therapy compared with pain intensity alone. Neuromodulation 24, 68–75. doi: 10.1111/ner.13212, PMID: 32592618

[ref65] Pirvulescu I. Biskis A. Candido K. D. Knezevic N. N. (2022). Overcoming clinical challenges of refractory neuropathic pain. Expert. Rev. Neurother. 22, 595–622. doi: 10.1080/14737175.2022.2105206, PMID: 35866187

[ref66] Puylaert M. Kapural L. Van Zundert J. Peek D. Lataster A. Mekhail N.. (2011). Pain in chronic pancreatitis. Pain Pract. 11, 492–505. doi: 10.1111/j.1533-2500.2011.00474.x 21676159

[ref67] Raja S. N. Carr D. B. Cohen M. Finnerup N. B. Flor H. Gibson S.. (2020). The revised International Association for the Study of Pain definition of pain: concepts, challenges, and compromises. Pain 161, 1976–1982. doi: 10.1097/j.pain.0000000000001939, PMID: 32694387 PMC7680716

[ref68] Rea A. G. Erdek M. A. (2007). Spinal cord stimulation for refractory angina pectoris and nonreconstructable chronic critical leg ischemia. Neurosurg. Q. 17, 208–214. doi: 10.1097/WNQ.0b013e318149e443

[ref69] Rigoard P. Ounajim A. Goudman L. Banor T. Héroux F. Roulaud M.. (2022). The challenge of converting “failed spinal cord stimulation syndrome” back to clinical success, using SCS reprogramming as salvage therapy, through Neurostimulation adapters combined with 3D-computerized pain mapping assessment: a real life retrospective study. J. Clin. Med. 11:23. doi: 10.3390/jcm11010272, PMID: 35012013 PMC8746025

[ref70] Rigoard P. Ounajim A. Goudman L. Bouche B. Roulaud M. Page P.. (2021). The added value of subcutaneous peripheral nerve field stimulation combined with SCS, as salvage therapy, for refractory low Back pain component in persistent spinal pain syndrome implanted patients: a randomized controlled study (CUMPNS study) based on 3D-mapping composite pain assessment. J. Clin. Med. 10:20. doi: 10.3390/jcm10215094, PMID: 34768614 PMC8584602

[ref71] Rigoard P. Ounajim A. Goudman L. Louis P. Y. Slaoui Y. Roulaud M.. (2021). A novel multi-dimensional clinical response index dedicated to improving global assessment of pain in patients with persistent spinal pain syndrome after spinal surgery, based on a real-life prospective multicentric study (PREDIBACK) and machine learning techniques. J. Clin. Med. 10:20. doi: 10.3390/jcm10214910, PMID: 34768428 PMC8585086

[ref72] Schechtmann G. Song Z. Y. Ultenius C. Meyerson B. A. Linderoth B. (2008). Cholinergic mechanisms involved in the pain relieving effect of spinal cord stimulation in a model of neuropathy. Pain 139, 136–145. doi: 10.1016/j.pain.2008.03.023, PMID: 18472215

[ref73] Schu S. Slotty P. J. Bara G. von Knop M. Edgar D. Vesper J. (2014). A prospective, randomised, double-blind, placebo-controlled study to examine the effectiveness of burst spinal cord stimulation patterns for the treatment of failed back surgery syndrome. Neuromodulation 17, 443–450. doi: 10.1111/ner.12197, PMID: 24945621

[ref74] Shealy C. N. Mortimer J. T. Reswick J. B. (1967). Electrical inhibition of pain by stimulation of the dorsal columns: preliminary clinical report. Anesth. Analg. 46, 489–491. doi: 10.1213/00000539-196707000-00025, PMID: 4952225

[ref75] Shechter R. Yang F. Xu Q. Cheong Y. K. He S. Q. Sdrulla A.. (2013). Conventional and kilohertz- frequency spinal cord stimulation produces intensity- and frequency-dependent inhibition of mechanical hypersensitivity in a rat model of neuropathic pain. Anesthesiology 119, 422–432. doi: 10.1097/ALN.0b013e31829bd9e2, PMID: 23880991 PMC3763697

[ref76] Soffin E Erdek M Raja SN, Eds. Interventional therapies for neuropathic Cancer pain. 15th world congress of pain clinicians; 2012 Jun 27–30; world Soc pain clinicians (WSPC), Granada, SPAIN. Medimond SRL (2012).

[ref77] Song Z. Y. Meyerson B. A. Linderoth B. (2011). Spinal 5-HT receptors that contribute to the pain-relieving effects of spinal cord stimulation in a rat model of neuropathy. Pain 152, 1666–1673. doi: 10.1016/j.pain.2011.03.012, PMID: 21514998

[ref78] Song Z. Meyerson B. A. Linderoth B. (2015). High-frequency (1 kHz) spinal cord stimulation—is pulse shape crucial for the efficacy? A pilot study. Neuromodulation 18, 714–720. doi: 10.1111/ner.12344, PMID: 26344573

[ref79] Sparkes E. Duarte R. V. Mann S. Lawrence T. R. Raphael J. H. (2015). Analysis of psychological characteristics impacting spinal cord stimulation treatment outcomes: a prospective assessment. Pain Physician 18, E369–E377. doi: 10.36076/ppj.2015/18/E369, PMID: 26000684

[ref80] Stiller C. O. Cui J. G. Oconnor W. T. Brodin E. Meyerson B. A. Linderoth B. (1996). Release of gamma-aminobutyric acid in the dorsal horn and suppression of tactile allodynia by spinal cord stimulation in mononeuropathic rats. Neurosurgery 39, 367–375. doi: 10.1097/00006123-199608000-00026, PMID: 8832675

[ref81] Su S. X. Shao J. P. Zhao Q. Z. Ren X. H. Cai W. H. Li L.. (2017). MiR-30b attenuates neuropathic pain by regulating voltage-gated Sodium Channel Nav1.3 in rats. Front. Molec. Neurosci. 10:15. doi: 10.3389/fnmol.2017.00126, PMID: 28529474 PMC5418349

[ref82] Treede R. D. Rief W. Barke A. Aziz Q. Bennett M. I. Benoliel R.. (2019). Chronic pain as a symptom or a disease: the IASP classification of chronic pain for the international classification of diseases (ICD-11). Pain 160, 19–27. doi: 10.1097/j.pain.0000000000001384 30586067

[ref83] Yang F. Zhang C. Xu Q. Tiwari V. He S. Q. Wang Y.. (2015). Electrical stimulation of dorsal root entry zone attenuates wide-dynamic-range neuronal activity in rats. Neuromodulation 18, 33–40. doi: 10.1111/ner.12249, PMID: 25308522 PMC4333027

[ref84] Yong R. J. Mullins P. M. Bhattacharyya N. (2022). Prevalence of chronic pain among adults in the United States. Pain 163, e328–e332. doi: 10.1097/j.pain.0000000000002291 33990113

[ref85] Youn Y. Smith H. Morris B. Argoff C. Pilitsis J. G. (2015). The effect of high-frequency stimulation on sensory thresholds in chronic pain patients. Stereotact. Funct. Neurosurg. 93, 355–359. doi: 10.1159/000438998, PMID: 26444968

[ref86] Zucco F. Ciampichini R. Lavano A. Costantini A. De Rose M. Poli P.. (2015). Cost-effectiveness and cost-utility analysis of spinal cord stimulation in patients with failed Back surgery syndrome: results from the PRECISE study. Neuromodulation 18, 266–276. doi: 10.1111/ner.12292, PMID: 25879722 PMC5029591

