# Supplementary material for: Research hotspots and trends on spinal cord stimulation for pain treatment: a two-decade bibliometric analysis
Source: Front Neurosci. 2023 May 25;17:1158712. doi: 10.3389/fnins.2023.1158712 (PMC10248081; doi:10.3389/fnins.2023.1158712)
Supplement: Supplementary file 1 [file Data_Sheet_1.docx]

**Supplementary contents**

Supplementary table 1. The top ten countries and institutions with the most citations.

Supplementary table 2. The population and GDP of the top ten countries with the most publications (Based on World Bank data 2021).

Supplementary table 3. The top ten funding agencies for the output of research.

Supplementary table 4. The top five countries and their publications in each country cluster.

Supplementary table 5. The top five institutions and their publications in each institution cluster.

Supplementary figure 1. Pictorial representation of the countrywide collaboration (Produced on Dycharts www.dycharts.com).

Supplementary table 1. The top ten countries/regions and institutions with the most citations.

| **Ranking** | **Country/Region** | **Frequency** | **Institution** | **Frequency** |
| --- | --- | --- | --- | --- |
| 1 | USA | 15744 | JOHNS HOPKINS UNIVERSITY | 3300 |
| 2 | ENGLAND | 5552 | KAROLINSKA INSTITUTE | 2825 |
| 3 | BELGIUM | 3800 | UNIVERSITY OF EXETER | 2318 |
| 4 | NETHERLANDS | 3763 | REGINA GENERAL HOSPITAL | 1710 |
| 5 | CANANDA | 3260 | CLEVELAND CLINIC | 1506 |
| 6 | SWEDEN | 2874 | VARESE REG HOSPITAL | 1367 |
| 7 | SWITZERLAND | 2794 | MONTREAL NEUROL HOSPITAL | 1353 |
| 8 | ITALY | 2778 | AXXON PAIN MED | 1351 |
| 9 | AUSTRALIA | 2339 | BRAFORD HOSPITAL | 1351 |
| 10 | SPAIN | 2301 | UNIVERSITY OF PLYMOUTH | 1271 |

Supplementary table 2. The population and GDP of the top ten countries/regions with the most publications (Based on World Bank data 2021).

| **Ranking** | **Country/Region** | **Publications** | **GDP (trillion$)** | **Population** | **GDP per capital ($)** | **Publications/GDP** | **Publications/Population** |
| --- | --- | --- | --- | --- | --- | --- | --- |
| 1 | USA | 749 | 23,315,080,560,000.00 | 331,893,745 | 70248.6 | 3.E-11 | 2.E-06 |
| 2 | ENGLAND | 138 | 3,131,377,762,925.95 | 67,326,569.00 | 46510.3 | 4.E-11 | 2.E-06 |
| 3 | NETHERLANDS | 120 | 1,012,846,760,976.73 | 17,533,044 | 57767.9 | 1.E-10 | 7.E-06 |
| 4 | BELGIUM | 92 | 594,104,177,539.53 | 11,592,952 | 51247.0 | 2.E-10 | 8.E-06 |
| 5 | GERMANY | 81 | 4,259,934,911,821.64 | 83,196,078 | 51203.6 | 2.E-11 | 1.E-06 |
| 6 | ITALY | 79 | 2,107,702,842,669.73 | 59,109,668 | 35657.5 | 4.E-11 | 1.E-06 |
| 7 | SWEDEN | 75 | 635,663,801,201.77 | 10,415,811 | 61028.7 | 1.E-10 | 7.E-06 |
| 8 | SPAIN | 58 | 1,427,380,681,294.55 | 47,415,750 | 30103.5 | 4.E-11 | 1.E-06 |
| 9 | CANADA | 56 | 1,988,336,331,717.42 | 38246108.0 | 51,987.9 | 3.E-11 | 1.E-06 |
| 10 | PEOPLES R CHINA | 53 | 17,734,062,645,371.40 | 1,412,360,000 | 12556.3 | 3.E-12 | 4.E-08 |

Supplementary table 3. The top ten funding agencies for the output of research.

| **Ranking** | **Funding agencies** | **Count** | **Percentage（%）** | **Country/Region** |
| --- | --- | --- | --- | --- |
| 1 | Medtronic | 123 | 8.84 | USA |
| 2 | United States Department of Health and Human Services | 68 | 4.89 | USA |
| 3 | Boston Scientific | 67 | 4.81 | USA |
| 4 | USA National Institutes of health | 67 | 4.81 | USA |
| 5 | St Jude Medical | 51 | 3.66 | USA |
| 6 | Nevro Corporation | 48 | 3.45 | USA |
| 7 | Abbott Laboratories | 24 | 1.72 | USA |
| 8 | Cational Natural Science Foundation of China NSFC | 14 | 1.01 | CHINA |
| 9 | National Institute of Neurological Disorders | 12 | 0.86 | USA |
| 10 | Dutch Government | 8 | 0.57 | Netherlands |

Supplementary table 4. The top five countries/regions and their publications in each country/region cluster.

| **Cluster label** | **Country/Region** | **Publications** |
| --- | --- | --- |
| #0failed back surgery syndrome | NETHERLANDS | 120 |
|  | BELGIUM | 92 |
|  | GERMANY | 81 |
|  | ITALY | 79 |
|  | SPAIN | 58 |
| #1surgery syndrome | USA | 749 |
|  | SOUTH KOREA | 25 |
|  | FINLAND | 16 |
|  | NEWZEALAND | 15 |
|  | BRAZIL | 14 |
| #2conventional medical management | ENGLAND | 138 |
|  | CANADA | 56 |
|  | PEOPLES R CHINA | 53 |
|  | POLAND | 11 |
|  | MEXICO | 3 |
| #3relieving effect | SWEDEN | 75 |
|  | SCOTLAND | 19 |
|  | TURKEY | 7 |
|  | NORWAY | 4 |
|  | LEBANON | 3 |

Supplementary table 5. The top five institutions and their publications in each institution cluster.

| **Cluster label** | **Institution** | **Publications** | **Cluster label** | **Institution** | **Publications** |
| --- | --- | --- | --- | --- | --- |
| #0multicenter retrospective study | Albany Medical Center | 59 | #6international multicenter study | Comprehens Pain Management Fox Valley | 5 |
|  | Cleveland Clinic | 47 |  | Oregon Hlth & Sci University | 5 |
|  | Mayo Clinic | 38 |  | Adv Bion Corporation | 4 |
|  | Harvard University | 35 |  | Comprehens Pain Center | 4 |
|  | Nevro Corporation | 32 |  | California Pain Medical Center | 3 |
| #1appropriate use | Johns Hopkins University | 60 | #7chronic back pain patient | Stanford University | 60 |
|  | Karolinska Institute | 58 |  | St Jude Med | 9 |
|  | Duke University | 33 |  | University of Otago | 8 |
|  | Ctr Pain Relief | 19 |  | University of Texas Dallas | 6 |
|  | Case Western Reserve University | 16 |  | Hunter Pain Clinic | 4 |
| #2conventional medical management | James Cook University Hospital | 24 | #8post-surgical complication | University of Eastern Finland | 10 |
|  | University of Exeter | 20 |  | University of Toronto | 9 |
|  | Aarhus University Hospital | 10 |  | University of Minnesota | 6 |
|  | Liverpool University | 9 |  | Thomas Jefferson University | 5 |
|  | Basildon & Thurrock University Hospital | 8 |  | Charles University in Prague | 4 |
| #3failed back surgery syndrome | Poitiers University | 42 | #9non-reconstructable critical ischaemia | Sahlgrens University Hospital | 6 |
|  | Ziekenhuis Brussel University | 32 |  | University of Amsterdam | 4 |
|  | VUB-Vrije Universiteit Brussel | 29 |  | Erasmus Universiteit Rotterdam | 3 |
|  | Maastricht University | 20 |  | Rijnstate Hospital | 3 |
|  | University of Valencia | 10 |  | Zucker School of Medicine Hofstra Northwell | 3 |
| #4long term safety | Carolinas Pain Institution | 23 | #11result | Advocate Illinois Masonic Medical Center | 4 |
|  | Boston Science Neuromodulation | 14 |  | Bluegrass Pain Consultants | 3 |
|  | Precis Spine Care | 8 |  | Capitol Pain Institution | 2 |
|  | Comprehens Pain & Rehabil | 7 |  | Univ Hosp Case Medical Center | 2 |
|  | Baylor College of Medicine | 6 |  | Bronson Neuroscience Center | 1 |
| #5physical activity | Iowa State University | 12 | #12precise study | Azienda Osped Univ Pisana | 4 |
|  | Illinois Wesleyan University | 8 |  | Azienda Osped Salvini | 2 |
|  | Natl Spine & Pain Center | 8 |  | Azienda Osped Santa Croce & Carle Cuneo | 2 |
|  | University of Virginia | 7 |  | Azienda Osped Univ Osped Circolo & Fdn Macchi | 2 |
|  | Medtronic | 7 |  | AORN SG Moscati | 2 |


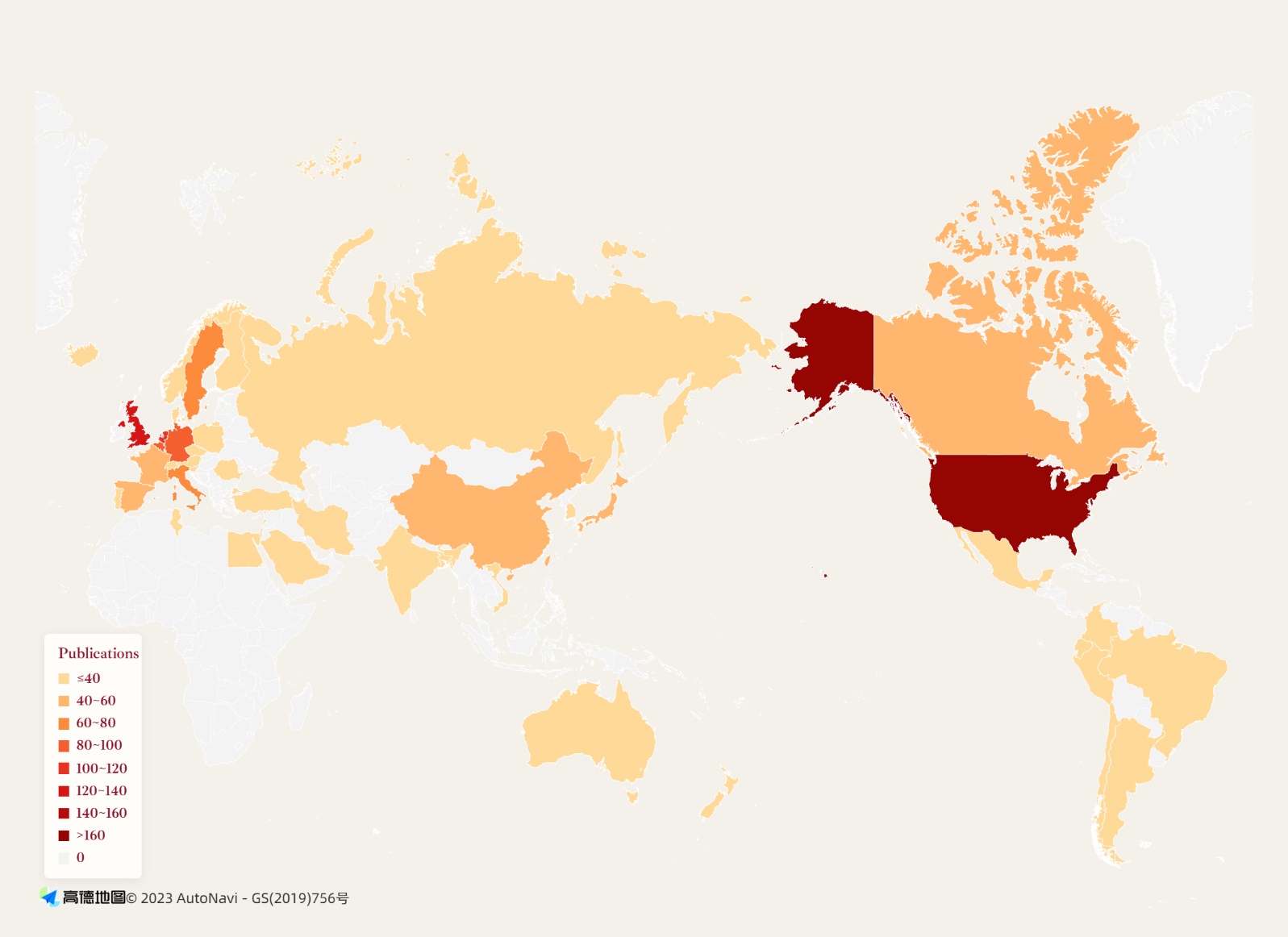


Supplementary figure 1. Pictorial representation of the countrywide collaboration (Produced on Dycharts [www.dycharts.com](http://www.dycharts.com)).
